# Resistive random access memory: introduction to device mechanism, materials and application to neuromorphic computing

**DOI:** 10.1186/s11671-023-03775-y

**Published:** 2023-03-09

**Authors:** Furqan Zahoor, Fawnizu Azmadi Hussin, Usman Bature Isyaku, Shagun Gupta, Farooq Ahmad Khanday, Anupam Chattopadhyay, Haider Abbas

**Affiliations:** 1 School of Computer Science and Engineering, Nanyang Technological University, Singapore, Singapore; 2 Department of Electrical and Electronics Engineering, Universiti Teknologi Petronas, Seri Iskandar, Malaysia; 3 School of Electronics and Communication Engineering, Shri Mata Vaishno Devi University, Katra, India; 4 Department of Electronics & Instrumentation Technology, University of Kashmir, Srinagar, India; 5 Division of Material Science and Engineering, Hanyang University, Seoul, South Korea; 6 School of Electrical and Electronic Engineering, Nanyang Technological University, Singapore, Singapore

**Keywords:** Resistive random access memory (RRAM), High-density memory, Power dissipation, Emerging memory technologies, 2D materials, Neuromorphic computing

## Abstract

The modern-day computing technologies are continuously undergoing a rapid changing landscape; thus, the demands of new memory types are growing that will be fast, energy efficient and durable. The limited scaling capabilities of the conventional memory technologies are pushing the limits of data-intense applications beyond the scope of silicon-based complementary metal oxide semiconductors (CMOS). Resistive random access memory (RRAM) is one of the most suitable emerging memory technologies candidates that have demonstrated potential to replace state-of-the-art integrated electronic devices for advanced computing and digital and analog circuit applications including neuromorphic networks. RRAM has grown in prominence in the recent years due to its simple structure, long retention, high operating speed, ultra-low-power operation capabilities, ability to scale to lower dimensions without affecting the device performance and the possibility of three-dimensional integration for high-density applications. Over the past few years, research has shown RRAM as one of the most suitable candidates for designing efficient, intelligent and secure computing system in the post-CMOS era. In this manuscript, the journey and the device engineering of RRAM with a special focus on the resistive switching mechanism are detailed. This review also focuses on the RRAM based on two-dimensional (2D) materials, as 2D materials offer unique electrical, chemical, mechanical and physical properties owing to their ultrathin, flexible and multilayer structure. Finally, the applications of RRAM in the field of neuromorphic computing are presented.

## Introduction

**Fig. 1:**
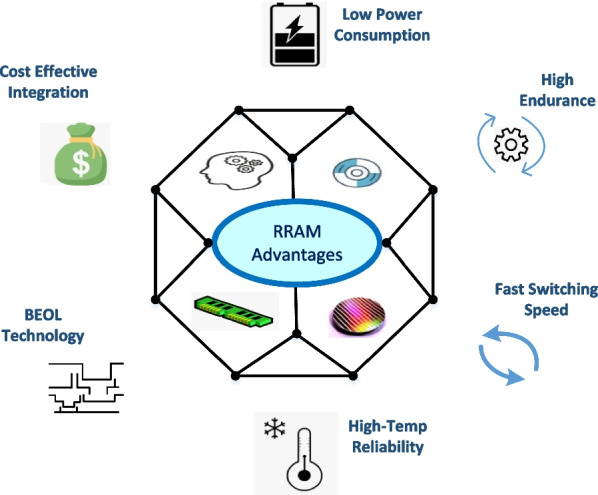
Advantages of RRAM

The domain of semiconductor electronics has witnessed significant growth during the last decade, and it continues to have significant influence on human society. This can be attributed to the unprecedented growth in the information communication technology field, as well as every other field of engineering and technology, thus increasing the demand for efficient information processing systems. The rapid growth of information technology systems has revolutionized products such as smart phones, miniaturized computers and Internet of things (IoT)-based devices, which requires high-performance computing technologies [1, 2]. Lately, composed entirely of electrical and mechanical components, products nowadays have become complex systems that combine hardware, data storage, sensors, software, microprocessors and connectivity in multiple ways. The conventional computing systems utilize Von Neumann architecture for performing computation tasks. In such systems, physically separated memory and computing units incur large latency and high energy consumption due to data shuttling which is commonly referred to as “Von Neumann Bottleneck” [3]. Since Moore’s law came into existence in the 1960s, the device computing ability has been enhanced by shrinking the electronic device size and facilitating packing densities of integrated circuits (ICs) at lower fabrication costs [4]. The significant roadblock for enhanced computation capability is the inherent drawback of the Von Neumann architecture due to the increasing gap between the CPU and memory. Thus, novel data processing technologies need to be explored to critically address the issue of insufficient computing capacities particularly in “memory” which nowadays constitutes about 60% of the processor area thus constituting for the major target of the designers for device miniaturization. Presently, researchers in nanoelectronics field are focusing their efforts on resistive random access memory (RRAM) which is one form of memristor technology as a feasible option for existing CMOS-based device miniaturization [5–13]. The research in RRAM continues to witness a tremendous growth as it is seen as the promising alternative to existing CMOS devices owing to its numerous advantages such as scalability, high data retention, CMOS and 3D integrability, multistate programmability, good endurance, lower power consumption and relatively high speed [14]. The main advantages of RRAM are highlighted in Fig. 1. The RRAM technology is approaching full-scale commercialization due to the modern-day device requirements of higher memory density with low power consumption, cost-effective fabrication, simpler manufacturing process and nonvolatile characteristics. Thus, the demand for this memory technology is growing and the interest is expected to increase in the coming years. RRAM is a resistive switching memory which covers a wide range memory and storage types of semiconductor devices [15]. In general, resistive switching memory includes any devices with resistance change under external stress. RRAM is essentially a resistive switch composed of a dielectric layer sandwiched between metal electrodes. The most striking feature of RRAM is that its conductance depends on the history of applied signals, thereby enabling it to function as a nonvolatile memory [16]. RRAM is also equipped with higher storage density as it can store multibit information due to its tunable conductance. The engineering efforts are mostly spent on how to better control the filament creation and switching in this emerging memory technology to improve the uniformity and stability.

### History of RRAM

**Fig. 2:**
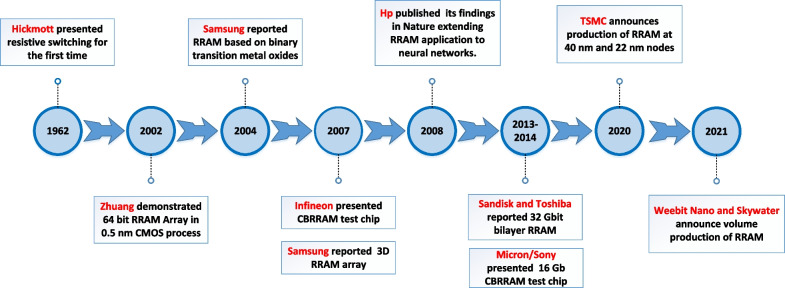
History of development RRAM from 1962 to 2021

The study of RRAM device initially began back in early 1960s, with the first reported work on resistive switching credited to Hickmott [17]. The resistive switching phenomenon at that time was reported in various oxide materials such as NiO, SiO_2_, Al_2_O_3_, TiO_2_, ZrO_2_, Ta_2_O_5_ and Nb_2_O_5_ [18–20]. However, in the following years, the research on resistive switching phenomenon did not pick up the pace. In the year 2000, the research on RRAM was lightened by the report from researchers at University of Houston [21] who observed the phenomenon of resistive switching in magnetoresistive films. This finding was significant as it brought a new boom in the field of RRAM research which was forgotten for long. In 2002, Zhuang et al. reported Pr_0.7_Ca_0.3_MnO_3_ -based 64-bit RRAM array using a 0.5-$\mu$m CMOS process line [22]. During the years 2004 to 2007, significant research efforts from the teams of Infineon and Samsung bore fruit, with the development of first 3D RRAM array demonstrated in 2007 [23]. Prior to that in 2004, Samsung demonstrated a simple RRAM based on binary transition-metal oxide fully integrated with 0.18 $\mu$m CMOS technology [24]. In 2008, Strukov et al. [25] from HP in its paper titled “the missing memristor found” published in Nature extended the use of RRAM for various applications and is thought of as the turning point which shifted the attention of researchers from across the globe significantly towards RRAM development. In 2010, Christophe et al. of unity semiconductor [26] successfully demonstrated the 64-MB prototype RRAM test chip with the promise for next-generation nonvolatile memory. In the following years, SanDisk/Toshiba demonstrated 32-Gb RRAM memory device in 24-nm technology [27], and Micron/Sony presented a 16-Gb RRAM prototype in 27-nm technology [28, 29]. In 2016, Qing et al. from Institute of Microelectronics, Chinese Academy of Sciences, reported the 3D vertical crossbar RRAM array with ultra-low power operation [30]. In 2020, TSMC announced the production of RRAM at 40-nm and 22-nm nodes [31]. The company in its statement said that RRAM allows us to scale to smaller geometries. It is a better scaling path than floating gate technology, particularly in terms of cost. In September 2021, Weebit Nano, a developer of next-gen memory technologies announced a deal with US-based technology foundry Skywater to bring RRAM technology into volume production [32]. ReRAM manufactured by Weebit Nano is said to be cost-effective, have enhanced endurance and retention at high temperature ranges, tolerant to radiation and electromagnetic fields and cause zero interference with front-end-of-line (FEOL) analog components. The most significant events in the history of RRAM development are detailed in the timeline graph shown in Fig. 2.

There have also been some key research breakthroughs from leading research institutes such as Institute of Microelectronics, Chinese Academy of Sciences, China, Tsinghua University, China, Interuniversity Microelectronics Centre (IMEC), Belgium, Stanford University, USA, etc., which has significantly contributed to the overall development of RRAM device technology over the recent years. In 2020 IEEE International Electron Devices Meeting (IEDM), research team led by Liu Ming from Institute of Microelectronics, Chinese Academy of Sciences, demonstrated the RRAM integration on the 14-nm FinFET logic process platform for the first time and realized the embedded RRAM memory chip of 1Mbit [33]. The research team demonstrated a design rule for integration of RRAM at sub-10 nm nodes. This work has a significant potential towards improvement in RRAM-embedded applications in advanced process nodes. In 2021, a team of researchers from Tsinghua University demonstrated a non-Markov chain algorithm in a two-dimensional (2D) mineral-based RRAM device. The findings of the study were reported in the journal Science Bulletin, and this also was a first attempt where 2D mica RRAM device was shown to exhibit unique non-Markov chain characteristic [34]. This work demonstrates significant potential of 2D mineral materials for electronics and further opens the door for the production of such RRAM devices with numerous functions and applications. In February 2022, article published in electronics weekly mentioned that IMEC had collaborated with Intrinsic Semiconductor Technologies to successfully scale its silicon oxide-based RRAM and demonstrated desirable characteristics, thus paving the way for the cost efficient, enhanced performance for nonvolatile memory in logic devices at advanced processing nodes for use in edge AI and IoT applications [35]. Recently, in August 2022, Stanford University Engineers presented a novel RRAM chip called “NeuRRAM”, which has AI processing capability within the memory, thus eliminating the need of having separate compute and memory units. This article which was published in Nature claims the chip to be of fingertip size, having more processing capabilities and less battery consumption than the current state-of-the-art chips [36].

The focus of this manuscript is to provide a review of research on various important aspects, including exploration of switching mechanisms, current performance metrics, investigation of materials employed for RRAM devices and the neuromorphic applications of RRAM. In this paper, we aim to coherently discuss all these aspects of RRAM technology, and it is mainly focused at providing readers a comprehensive reference for future investigation and development of low power and highly scalable RRAM devices. “RRAM design and physical mechanism” and “Switching mechanism of RRAM” sections highlight details of switching mechanisms of RRAM together with the classification of switching modes. The key figures of merit for RRAM devices are detailed in “Figures of merit of RRAM devices” section. “Three-dimensional (3D) integration of RRAM” section discusses various materials employed for the RRAM design with emphasis primarily on 2D materials. “Three-dimensional (3D) integration of RRAM” section addresses the applications of RRAM to the field of neuromorphic computing. The “Summary and outlook” section concludes the paper.

## RRAM design and physical mechanism

**Fig. 3:**
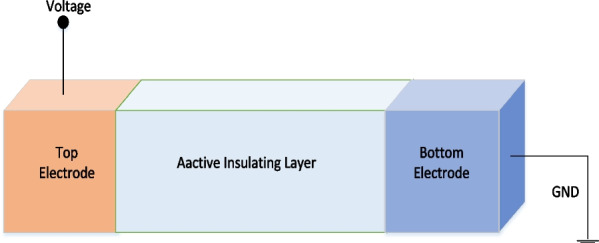
Schematic of metal–insulator–metal structure for RRAM

The device structure of RRAM is simple capacitor-like metal–insulator–metal (MIM) structure with switching layer sandwiched between two metal electrodes. The schematic of the RRAM cell is shown in Fig. 3. The resistance of the MIM structure can be changed on the application of proper electrical signal, and the device retains the current resistance state until an appropriate signal is applied to vary its resistance representing the nonvolatile nature of the device [37, 38]. Due to the simple structure of the RRAM device, it can be integrated easily in passive crossbar arrays with a small size of 4F$^{2}$ (F is the minimum feature size), and the size can be further reduced to 4F$^{2}$/n within vertically stacked three-dimensional (3D) architectures (n is the stacking layer number of the crossbar array) [39].

**Fig. 4:**
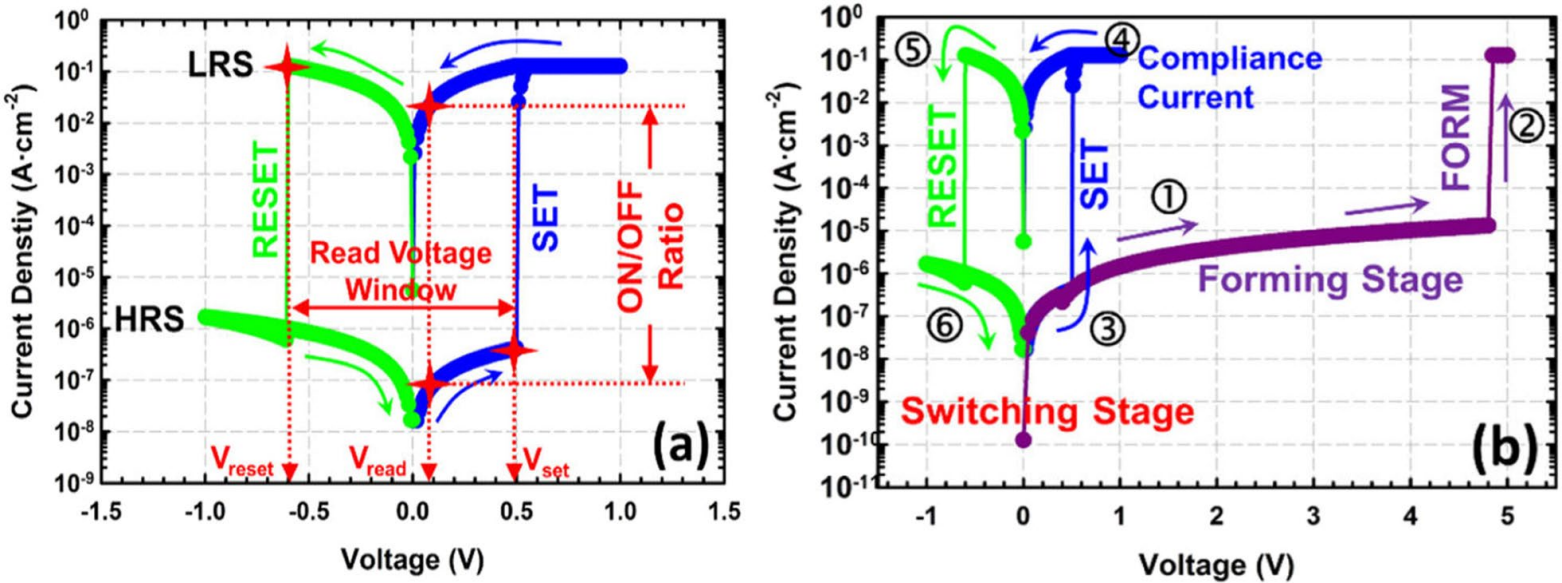
**a** Plot of current–voltage of RRAM. **b** Typical I–V characteristics with forming stage included [15]

In RRAM, device resistance is varied by the application of the external voltage pulse across the electrodes. The intrinsic physical phenomenon behind RRAMs is resistive switching (RS), which means that the device can be freely programmed into a high-resistance state (HRS, or OFF state) or a low-resistance state (LRS, or ON state) under external electrical stimuli. The conventional memory storage devices store data in binary form “0” and “1”, where “0” represents the data that are not stored and “1” represents the stored data [40, 41]. RRAM devices utilize redox reactions ( oxidation and reduction ) for effective data storage wherein redox reactions form a conducting filament (CF) between the two metal electrodes, within the insulator. Due to the application of external electric pulse, the filament is formed between the two metal electrodes of RRAM and the device is said to be in the low-resistance state (LRS) usually referred to as logic state “1”. When the filament is ruptured, the device is said to be in the high-resistance state (HRS) usually referred to as logic state “0” [42]. The schematic flow diagram depicting the operating mechanism of the RRAM is shown in Fig. 4.

**Fig. 5:**
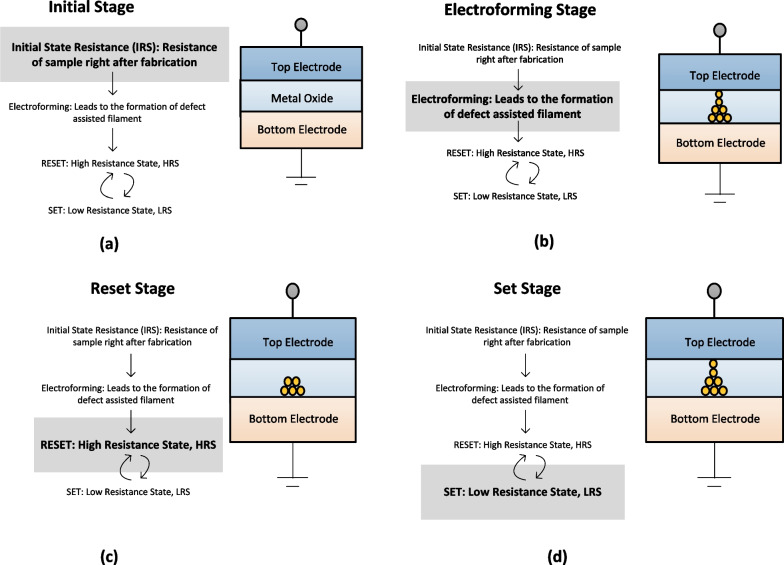
Description of the switching stages of RRAM

Initially, the RRAM is in its pristine HRS, to switch the device from its initial HRS to LRS, the application of the electrical pulse signal enables the formation of conductive paths in the switching layer and the RRAM cell is switched into LRS. This is referred to as “forming” or “electroforming” process of the RRAM, and the voltage at which this process occurs is referred to as forming voltage ($V_{f}$) [43]. To enable switching transition of RRAM from LRS to HRS, RESET voltage ($V_{\rm reset}$) is applied and the process is referred to as the “RESET” of the RRAM device. The HRS of the RRAM can be changed to LRS on the application of the voltage pulse. The voltage at which the transition occurs from HRS to LRS is referred to as SET voltage ($V_{\rm set}$) and the process is referred to as the “SET” process. Thus, resistance states in RRAM are obtained by switching between the LRS and HRS on the application of set voltage ($V_{\rm set}$) and reset voltage ($V_{\rm reset}$), respectively, as depicted in Fig. 4a. During the SET operation, the current limit, called the compliance current ($I_{cc}$), protects the device from an uncontrolled CF formation and avoids permanent damage to the device [44]. However, some RRAMs can exhibit self-compliance during the SET operation, eliminating the need for an extra circuitry for the current compliance [45]. To achieve enhanced storage density, multilevel cell capability in RRAM is observed and can be realized by adopting different methods such as varying the compliance current, sweep rate and the reset voltage. Figure 4b depicts the current–voltage (I–V) characteristics of RRAM with forming voltage applied during the forming stage of RRAM. The sweeping sequence of the switching stage is indicated by the numbers beside arrows. The description of the switching stages of RRAM is presented in Fig. 5.

Based on the applied voltage polarity, RRAM has the following switching modes: (i) unipolar switching and (ii) bipolar switching. In unipolar switching mode, the operation of RRAM depends upon the magnitude of the applied voltage. The set and the reset operations of RRAM occur at voltages of same polarity but different magnitudes. For bipolar switching mode, the critical requirement to perform the switching (set and reset) operation is the use of voltages of opposite polarities. In other words, the transition from a HRS to LRS occurs at either voltage of positive polarity or negative polarity and the voltage of opposite polarity to that of the applied voltage for LRS switches the RRAM cell back into its HRS [46]. The most relevant explanation for unipolar switching of RRAM is the formation and the disruption of the conductive filament with Joule heating effect as the main driving factor for the resistance change. Thus, in unipolar mode, the set/reset transitions of RRAM are achieved by thermally induced formation and rupture of the filaments in the resistive switching oxide layer. The bipolar mode of operation for RRAM is mostly associated with nano-ionic transport mechanism which utilizes the redox equilibrium mainly driven by an external field. For bipolar RRAM, the switching speed depends upon the several electrochemical kinetics that are responsible for the formation and rupture of conductive metallic filaments.

It has been reported lately that various RRAM devices exhibit both bipolar and unipolar resistive switching within the same device; such switching mechanism is referred to as nonpolar or mixed bipolar/unipolar. The RRAM devices of such nature have the compliance current as the significant factor for determining the bipolar operating mode (usually at low current) or unipolar mode (usually at high current due to Joule heating). Various oxide materials such as ZrO$_{x}$ [47, 48], TiO$_{x}$ [49, 50], AlO$_{x}$ [51, 52] and HfO$_{x}$ [53, 54] are reported to exhibit such behaviour. The exact reason for this co-existent unipolar and bipolar behaviour is not yet understood clearly but can possibly be explained in terms of the formation and rupturing of conductive filaments.

## Switching mechanism of RRAM

A variety of switching mechanisms can be observed in RRAM based on their physical phenomena which mainly depend upon the materials employed and the fabrication processes. The switching in RRAM is attributed to the formation and the rupture of CF within the oxide layer, causing the resistance shift in device from the “Off” state to the “On” state and vice versa [55, 56]. For understanding resistive switching mechanism of RRAM devices, three different types of classifications exist: (i) The first type is based on migration of anions, where oxygen vacancies contribute to the conductive path within the oxide layer. This classification is usually referred to as oxide-based RRAM (OxRAM) or valence change memory (VCM). (ii) The second classification is based on the formation of conductive paths formation via metal cations under an externally applied field. This type of RRAM is usually referred to as conductive bridge random access memory (CBRAM) or the electrochemical metallization (ECM) memory. (iii) The third classification is based on charge trapping/detrapping within the device, referred to as the electronic mechanism.

### Valence change memory (VCM) switching mechanism

**Fig. 6:**
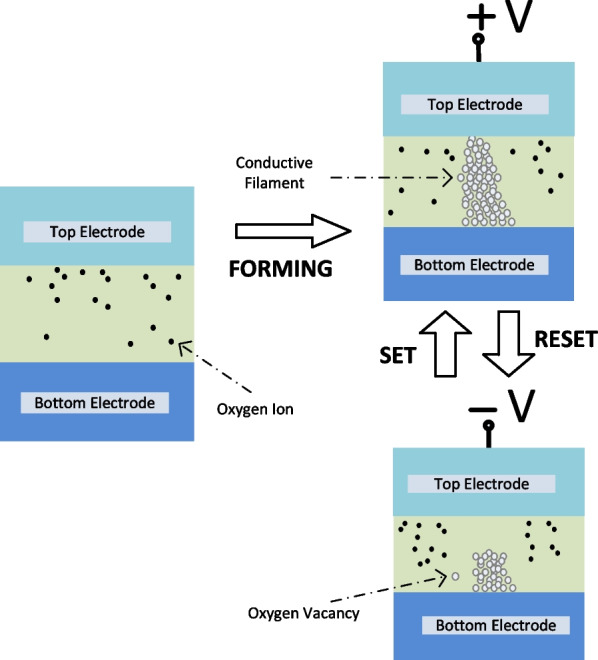
Schematic illustration of switching mechanism of VCM

In most cases, resistive switching in RRAM is caused by the migration of oxygen ions in the resistive switching layer sandwiched between the bottom electrode and the top electrode of the RRAM device, leading to the formation of the conductive paths referred to as CFs [57–59]. The schematic diagram of the switching mechanism of the VCM-based RRAM device depicting the oxygen ion migration and diffusion is depicted in Fig. 6. Initially, during the forming process the oxygen ions move towards the anode interface due to the soft dielectric breakdown caused by the high electric field leaving behind oxygen vacancies in the resistive switching layer. Thus, defects are generated, which results in the formation of CFs [60, 61]. For transition of the memory cell from LRS to HRS, reset process occurs during which the oxygen ions migrate back to the bulk to recombine with the oxygen vacancies. The resistance switching in VCM-based RRAM devices can be modulated by further tuning the initially formed conductive filaments by controlling the magnitude of external electric field applied [62–64]. The precise explanation of the filament formation due to the application of the electric field assisted with Joule heating is still one of the major complexities to unravel in the valence change model.

### Electrochemical metallization memory (ECM) switching mechanism

In some devices, the switching mechanism is based on movement of metallic ions triggered by externally applied electric field; such devices are commonly referred to as electrochemical metallization RRAMs (ECM-RRAMs) or conductive bridge RRAMs (CBRAMs) [65, 66]. The switching mechanism of ECM-based RRAM device depends on the oxidative interfacial dissolution of an active metal electrode, followed by subsequent cation migration across an ion-conducting electrolyte layer, acting as an insulator [67]. In such type of RRAMs, the MIM consists of an electrochemically active metal electrode such as Cu or Ag and an inert counter electrode such as Pt, W or Au [68–70]. Similar to VCM-based RRAM devices, electrochemical switching in ECM-based RRAM devices is based on filament formation throughout the insulator material acting as solid electrolyte. The formation of conductive channels is due to dissolved metal cations migration into the insulator region from the interface of the electrochemically active electrode. In the SET operation, the metallic ions from the active electrode diffuse into the insulator and get reduced on reaching the inert electrode, thus forming a CF [71]. In the RESET operation, the metallic atoms in the CF get oxidized, thus rupturing the CF and obstructing the flow of current.

**Fig. 7:**
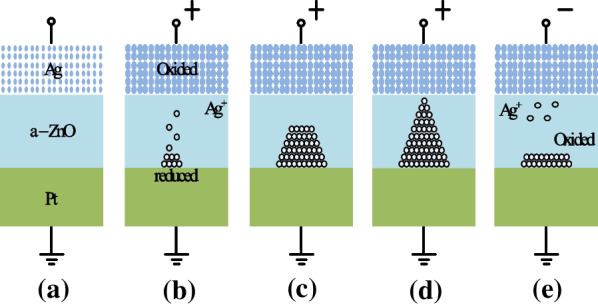
Schematic of the switching mechanism of conductive bridge RRAM: **a** Initial state. **b**, **c** Oxidation of Ag and migration of Ag$^{+}$ cations towards cathode and their reduction. **d** Accumulation of Ag atoms and Pt electrode leads to growth of highly conductive filament. **e** Filament dissolution takes place on applying voltage of opposite polarity [72]

To clearly depict the switching mechanism of CBRAM based RRAM, example of Ag/a-ZnO/Pt RRAM cells [72] is considered. The schematic depiction of switching mechanism for Ag/a-ZnO/Pt RRAM device is presented in Fig. 7. Figure 7a depicts the initial state of the ECM or CBRAM memory cell. The Ag top electrode (TE) is an active component in the filament formation, while the bottom Pt electrode is inert. Due to the positive bias at the Ag top electrode, the oxidation ( Ag$\rightarrow$ Ag$^{+}$ + e$^{-}$) occurs due to which Ag$^{+}$ cations are generated and then deposited into the switching layer (a-ZnO). The Pt bottom electrode (BE) attracts the Ag+ cations, due to its negative bias, due to which the reduction ( Ag$^{+}$ + e$^{-}$ $\rightarrow$ Ag ) occurs. Thus, Ag$^{+}$ cations are reduced to Ag atoms and accumulate until the conducting bridge is formed (Fig. 7b–d) and the RRAM device is said to exhibit LRS. This process is referred to as the “SET”. The application of negative voltage causes the CF to dissolve, and the device is said to be in the high-resistance state (HRS). This process is referred to as “RESET” and is depicted in Fig. 7e.

**Fig. 8:**
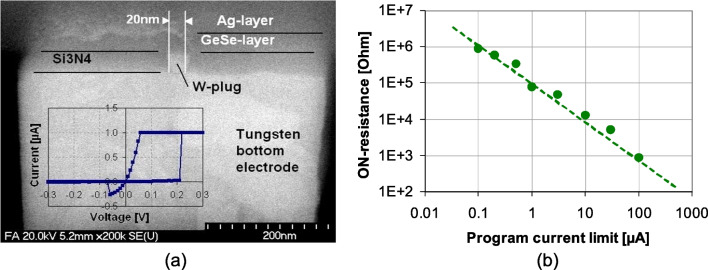
**a** SEM and IV curve of CBRAM **b** dependence of the ON-state resistance on programming current for CBRAM devices at room temperature [57]

Figure 8a shows SEM cross-sectional image and corresponding IV curve of RRAM device generated by deposition and etching of a dielectric layer ${\text {Si}}_{3}{\text {N}}_{4}$ on top of tungsten bottom electrode and filling the respective small via diameter (20 nm) with a tungsten plug. Finally, the thin GeSe chalcogenide layer and the silver top electrode are deposited and patterned. The structure as small as 20 nm exhibits clear switching characteristics. Figure 8b depicts the dependence of the ON-state resistance on the programming current in the 100-nA to 100-$\mu$A range.

### Electronic switching mechanism

In RRAM devices, the CF formation is mostly attributed to the ion migration and redox process. However, there are some cases of RRAM devices, in which the resistive switching is based on the electronic mechanism. Such resistive switching devices are based on charge trapping/detrapping mechanism. In Pt/${\text {Pr}}_{0.7}$ ${\text {Ca}}_{0.7}$ ${\text {MnO}}_{3}$/Ag RRAM, it was reported that some devices show both hysteretic and asymmetric behaviours in current–voltage characteristics. The observed conduction characteristics exhibit the space charge limited conduction (SCLC) effect, and the hysteretic behaviour can be ascribed to a carrier trapping and detrapping of the trap sites in the manganite [73].

## Figures of merit of RRAM devices

The RRAM characteristics are obtained employing a wide variety of materials with different switching mechanisms; thus, the characteristics of RRAM may vary widely. There are a few indices which serve as a measure of the performance of RRAM such as switching speed, endurance, retention, uniformity and scalability. In this review, we discuss briefly various figures of merit of RRAM devices.

### Switching speed

The write and read speed is one of the most significant performance indicators in terms of the figure of merit of the memories. The slower nature of the flash memory compared to the static random access memory (SRAM) is attributed to the slow charging rate of the floating gate over a large electronic barrier via electron tunnelling. Although SRAMs are faster, they are expensive and have large-area footprint. The RRAMs have demonstrated sub-100 ps switching speed [74] making them a suitable candidate for a variety of applications in near future. The factors that determine the switching mechanism (thus the switching speed) in RRAM are debatable; however, there are some known factors on which the switching speed of RRAM depends. The mobility of mobile ions related to either migration of metal cations or oxygen anions is considered one of the factors that determine the switching speed of RRAM. In RRAM, with an increase in electric field, the mobility of the ions increases exponentially, thus affecting the ion transport process. Temperature also affects the switching process in RRAM in terms of both the ion migration and the formation of the chemical bonds. The application of higher voltage generally enables the device to switch exponentially faster [75], but there are some undesirable effects of applying higher voltages specifically in terms of power consumption and device reliability.

**Fig. 9:**
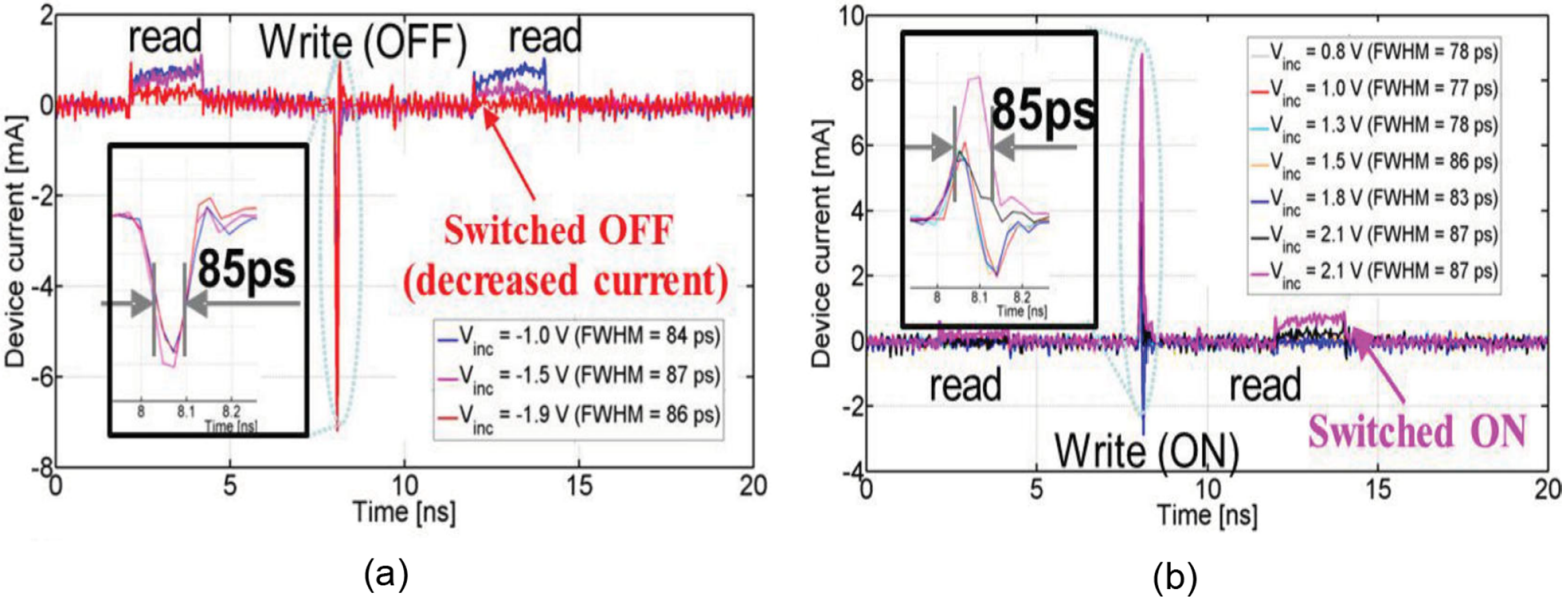
**a** 85-ps voltage pulse OFF Switching. **b** 85-ps voltage pulse ON Switching. FWHM means full width at half maximum. Reproduced with permission from [74]

In the work presented by Choi et al., switching speed of 85 ps was reported in a TiN/AlN/Pt device [74] as depicted in Fig. 9. The insets to Fig. 9a, b depict the zoom-in image of the switching pulses. As shown in the figure, the read pulse was applied before and after the switching pulse. The devices were switched successfully only if the threshold voltage was reached ($-$1.9 V for OFF switching and 2.1 V for ON switching). Choi et al. used nitride vacancies instead of the oxygen vacancies as the migration species owing to the fact that nitride vacancies have a valence of +3, which might be subjected to a stronger electric force compared to +2 valued oxygen vacancies. Since multiple steps are involved in switching process of CBRAM, such as ion transport, oxidation and reduction, attaching and detaching of ions from the electrode, this makes CBRAM switching slower. Thus, it takes some time before a stable electrode is able to bridge two electrodes [76]. Although slower, switching in CBRAM can still be completed within a few nanoseconds, as demonstrated experimentally [77].

### Endurance

In RRAM, the endurance is defined as the number of set/reset cycles a memory device can undergo while maintaining a distinguishable resistance ratio between them. Since the resistance ratio of the device degrades with increasing program/erase cycles, the device is said to have failed when the ON/OFF current ratio reaches below a certain threshold. There are various factors that have an impact on the RRAM device endurance with major ones being the material, processing, device structure and the electrical operation schemes. After repeated set/reset transitions, the device undergoes an irreversible change of the switching material at the active region of the device, forcing it to get stuck in its HRS or its LRS state [78]. During the cycling, in general, the HRS resistance tends to decrease, and usually, the final failure state of RRAM cells is stuck with LRS and unable to reset back to HRS. This can be caused by too many defects such as oxygen vacancies accumulated during the cycling in several ways: (i) too many oxygen vacancies generated at or near the electrode–oxide interface; (ii) too many oxygen vacancies in or near the filament; and (iii) too many oxygen vacancies in the oxide matrix.

**Fig. 10:**
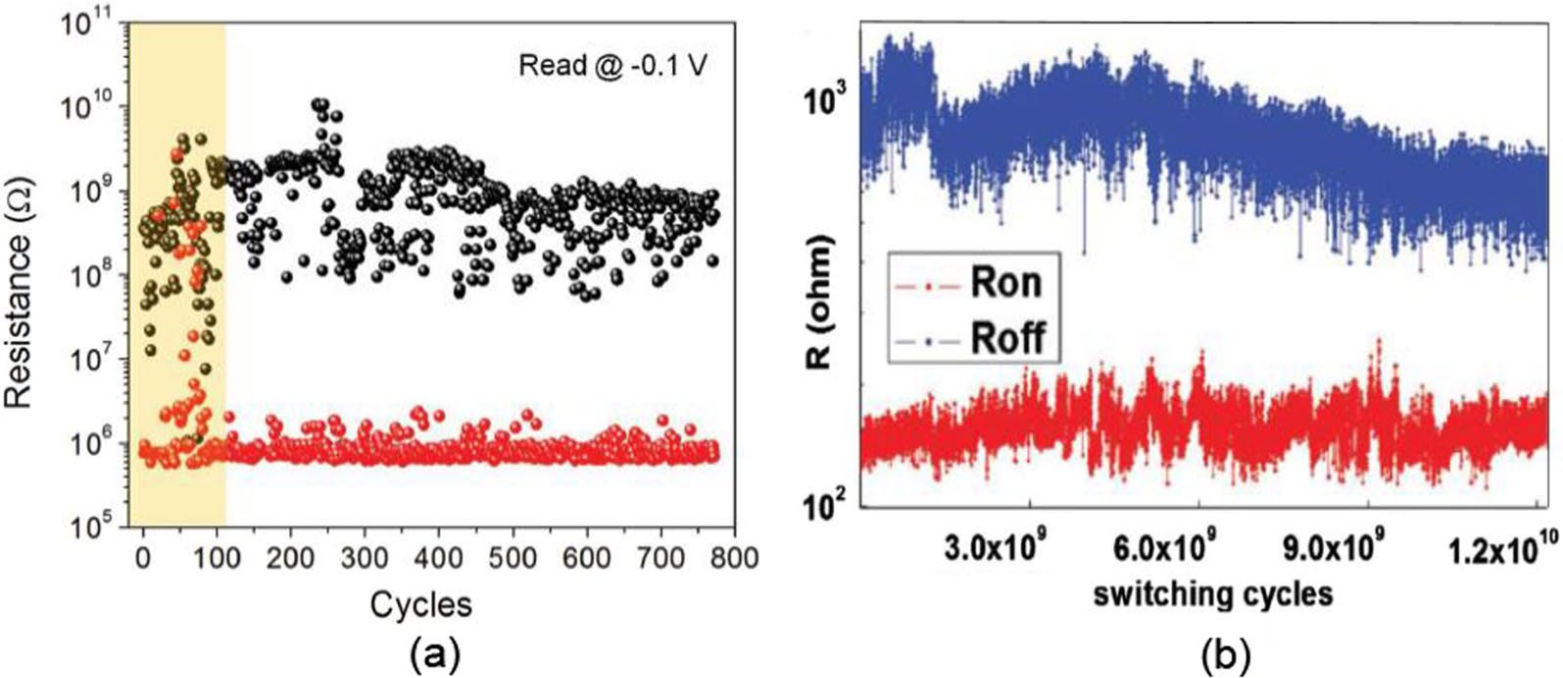
RRAM endurance plots obtained by two methods. **a** Measuring various I–V curves and extracting resistance at $-$0.1 V. Reproduced with permission [79] Copyright 2017, Wiley-VCH. **b** By using pulse stresses recording the current simultaneously and calculating the resistance for each cycle (namely current-visible PVS). Reproduced with permission from [80]. Copyright 2010, American Institute of Physics

Thus, an endurance test helps to determine the maximum number of set/reset cycles for the resistive switching device with distinguishable resistance ratio between the resistance of the high-resistance state ($R_{\rm HRS}$) and resistance of the low-resistance state ($R_{\rm LRS}$) as shown in Fig. 10. There are various methods of obtaining the endurance characteristics; however, the two most common methods are: (i) I–V sweeps and (ii) current-visible pulsed voltage stress (PVS). The first method of obtaining endurance involves the collection of sequences of I–V sweeps of the switching device and the subsequent extraction of $R_{\rm HRS}$ and $R_{\rm LRS}$ dividing a select read voltage (typically ± 0.1 V) by the corresponding currents at that voltage as shown in Fig. 10a [79]. Although this method of determining the resistance is highly reliable, the drawback of this method is that it is very slow as it takes long time ($\sim$30–60 s) for measuring I–V sweeps. In current-visible PVS method of measuring endurance, it involves applying a series of PVS to the device, in which the user can modify the voltages ($V_{\rm UP}$ and $V_{\rm DOWN}$) and simultaneously measure the currents driven [80]. To set/reset the device, a voltage pulse is applied and to read the conductance of the device, a read voltage of 0.1 V is applied after each stress. Similar to the I–V sweep method, the values of $R_{\rm HRS}$ and $R_{\rm LRS}$ are calculated for all test cycles as shown in Fig. 10b. This method of measuring device endurance matches well with the functioning of the realistic devices and is faster compared to the I–V sweeps, as the pulse widths can be of the order of microseconds, which allows collecting millions of cycles in few minutes of time duration. Currently, RS devices with endurance limits of up to 10$^{12}$ cycles have been reported in different types of MIM cells, including Pt/${\text {Ta}}_{2}{\text {O}}_{5-{\text {X}}}/{\text {TaO}}_{2-{\text {X}}}/{\text {Pt}}$ [81] and Ta/Ta${\text {O}}_{{\text {x}}}/{\text {TiO}}_{2}/{\text {Ti}}$ [82].

**Fig. 11:**
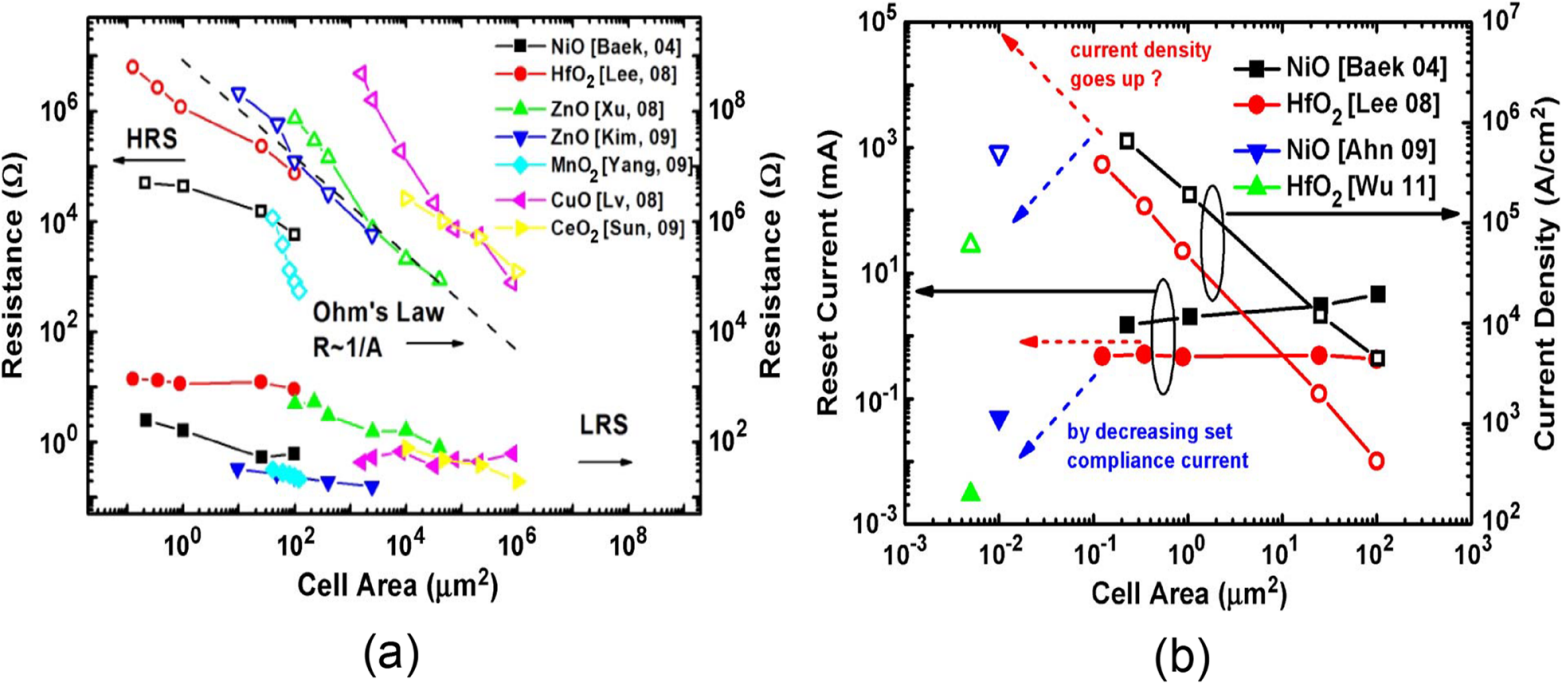
**a** HRS and LRS resistance vs cell area. **b** The peak value of reset current and corresponding current density versus cell area. Reproduced with permission from [84] Copyright IEEE

To improve the endurance performance in CBRAM devices, Zhao et al. suggested to localize cation injection into the RS layer through nanohole of inserted ion barrier between active electrode and switching layer of the device. An improved endurance was reported in the Cu/nanohole graphene/Hf$O_{2}$/Pt RRAM device due to the cation injection being limited by nanohole graphene. Due to this cation injection, some of the cations get oxidized from the active metal electrode and thus get injected into the RS layer only through the nanohole of the ion barrier instead of the whole active electrode area. This localized injection of cations form the active metal electrode into RS layer helps in reducing the random CF nature, thus resulting in an improved device performance of the CBRAM. The use of graphene as an ion barrier was preferred due to its excellent impermeability to ions and ease of fabricating nanoscale holes [83].

### Scalability

The development of RRAM device technology is motivated by its remarkable scalability potential to the nanometer regime [84]. Lee et al. reported the size of CF can be lower than 10 nm in NiO-based memory [85], thus demonstrating the potential of RRAM to scale to sub-10-nm dimensions. In addition to the top-down fabrication approach, the resistive switching behaviour is seen in the self-assembly grown metal oxide nanowires [86, 87], thereby demonstrating the scalability of RRAM to nanometer regime. Figure 11a depicts the plots of cell area vs resistance (HRS and LRS), thus demonstrating the scaling trends of the various RRAM devices. The resistance of HRS increases as the inverse of the cell area, roughly following the Ohm’s law, whereas the resistance of LRS has only a slight dependency on the cell area. This increasing HRS/LRS resistance ratio with a decrease of cell area is one of the factors which favours device scaling. The maximum current during the reset process defined as the reset current is another significant parameter as the peak power consumption is determined mostly by reset current. The reset current for the single memory cell is of the order of mA or hundreds of $\mu$A. The general scaling trends of the reset current and the corresponding reset current density for various RRAM devices are reported in [82, 88]. It is observed that the reset current slightly decreases when scaling down RRAM devices, thus causing an appreciable increase of current density required for reset. This issue can be addressed by utilizing a smaller set compliance current during the set process, because the reset current is almost linear with the set compliance current, as depicted in Fig. 11b. The precise basis for the linear set and reset current relation is that the small set compliance current causes weak CFs formation which only require a smaller reset current for the rupture process. It is observed that the LRS resistance becomes higher because of smaller set compliance current. Fortunately, in RRAMs, the reset current is independent of the cell area but depends on the set compliance current. Thus, in smaller area cells, smaller set compliance can be used; therefore, the reset current can scale down with device size.

### State retention

The time period for which a memory device can maintain its programmed resistance state (LRS and HRS) at a certain temperature is referred to its “retention”. In other words, retention is defined as the length of time for which the RRAM resistance states (LRS and HRS) remain stable after the SET/RESET operations [89]. Various studies on RRAM have concluded based on experimental observations that retention in the LRS strongly depends on the compliance current used during the SET operation [90]. It is also observed that retention time degrades at higher temperatures due to the possibility of frequent atomic rearrangements [91]. The retention characteristics of RRAM are measured by applying a constant voltage stress (CVS) over time after inducing the set/reset transition (usually by an I–V sweep) and using a low read voltage (±0.1V) and extracting the current versus time (I–t) curve for each resistive state [92]. For nonvolatile memory applications, data retension time longer than ten years is expected. The retention time must be maintained at thermal stress up to $85^\circ$ C and small electrical stress such as constant stream of READ pulses. In RRAMs, long retention time in LRS is a challenge because the atomic rearrangements induced during the set transition may weaken over time. In HRS, on the other hand the retention is not that significant a concern because of the fact that HRS is normally the natural state of the RRAM device and the device will continue to remain in the HRS if low or no bias is applied.

**Fig. 12:**
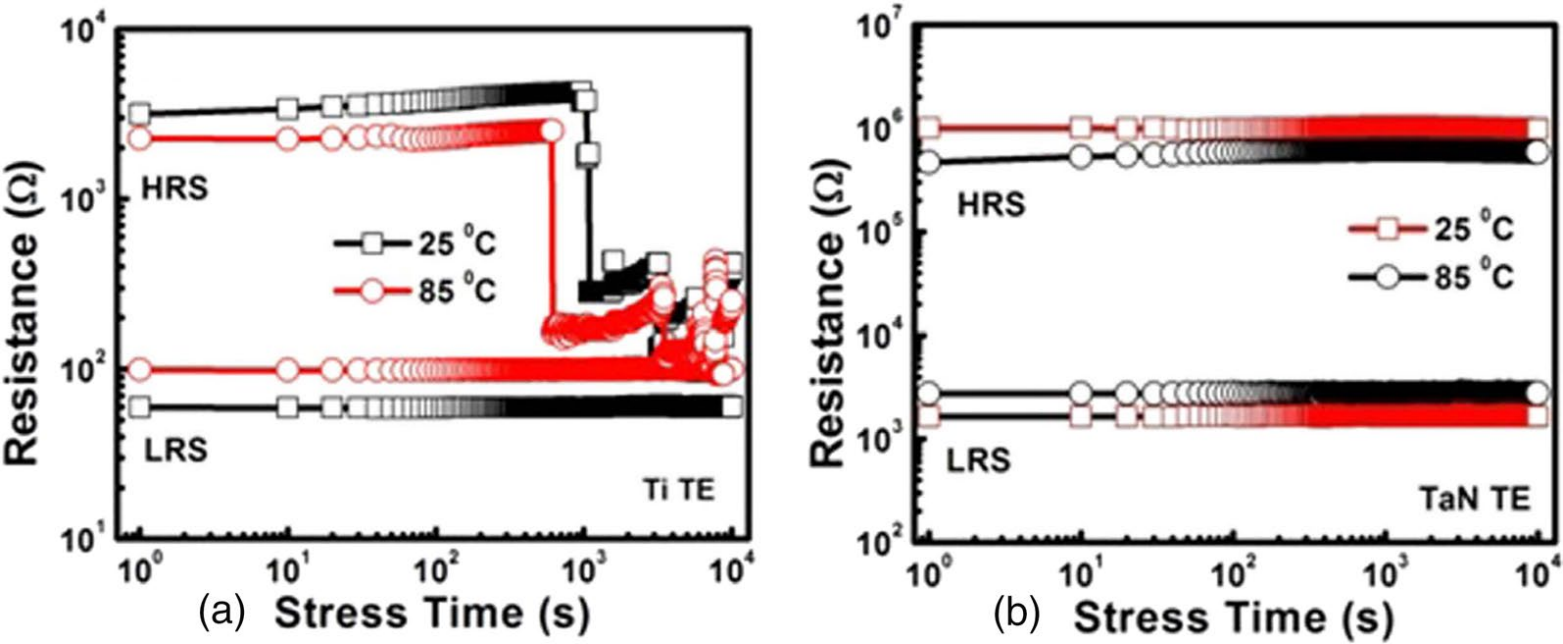
Retention characteristics **a** Ti top electrode. **b** TaN top electrode. Reproduced with permission from [93]

Anwar et al. [93] reported the state retention or the data retention characteristics of Ce$O_{2}$/Ti/Ce$O_{2}$ trilayered films sandwiched between Pt bottom electrode and two different top electrodes (Ti and TaN). To test the retention characteristics, both the Ti and TaN top electrode-based devices were transitioned from HRS to LRS (SET cycle) and then the electrical stress of +0.2V was applied for 10$^{4}$s with 10 s-time interval at room temperature and $85^\circ$ C. The process was repeated for the RESET cycle with negative electrical stress of $-$0.2 V was applied for 10$^{4}$s both at room temperature and 85 degree Celsius. From Fig. 12a, for Ti top electrode-based RRAM device, the results demonstrate the failure of the HRS characteristics after 10$^{2}$s observed at room temperature and $85^\circ$ C. The reason for this failure in data retention characteristics is attributed to the thicker interfacial TiO layer, due to the inadequate rupture of too thick CFs during the RESET process. From Fig. 12b, for TaN top electrode-based RRAM device, a good stability is exhibited in the LRS and HRS with no major deterioration during the stress time both during room temperature and $85^\circ$ C. The improved retention characteristics in this case are attributed to the thinner interfacial TaON layer which acts as an oxygen diffusion barrier [94].

The retention performance of the RRAM device also depends upon the values of compliance current employed, which is usually set to prevent the permanent dielectric breakdown of the switching medium. The Si$O_{2}$ -based RRAM demonstrated both volatile and nonvolatile behaviours for two different compliance current values. The volatile behaviour was observed for 10 $\mu$A compliance current, whereas for 500 $\mu$A compliance current, the device exhibited nonvolatile behaviour [95]. Although the volatile and the nonvolatile behaviour of the device has been attributed to the low and high values of compliance currents, respectively, the impact of the intermediate compliance currents on the retention characteristics was not investigated much. In 2021, Khaled et al. [96] demonstrated the impact of intermediate compliance current on the performance of Cu/Hf$O_{2}$ -based RRAM device. Similar to the findings published previously, the experimental results showed that nonvolatile behaviour with high retention time was observed for higher compliance current values. Additionally, it was observed that intermediate compliance currents can control the retention time of the device during the programming step. The study also demonstrated high cycle-to-cycle variability in the retention time, thus providing good source of stochasticity which can be quite beneficial for hardware security applications.

### Uniformity

**Fig. 13:**
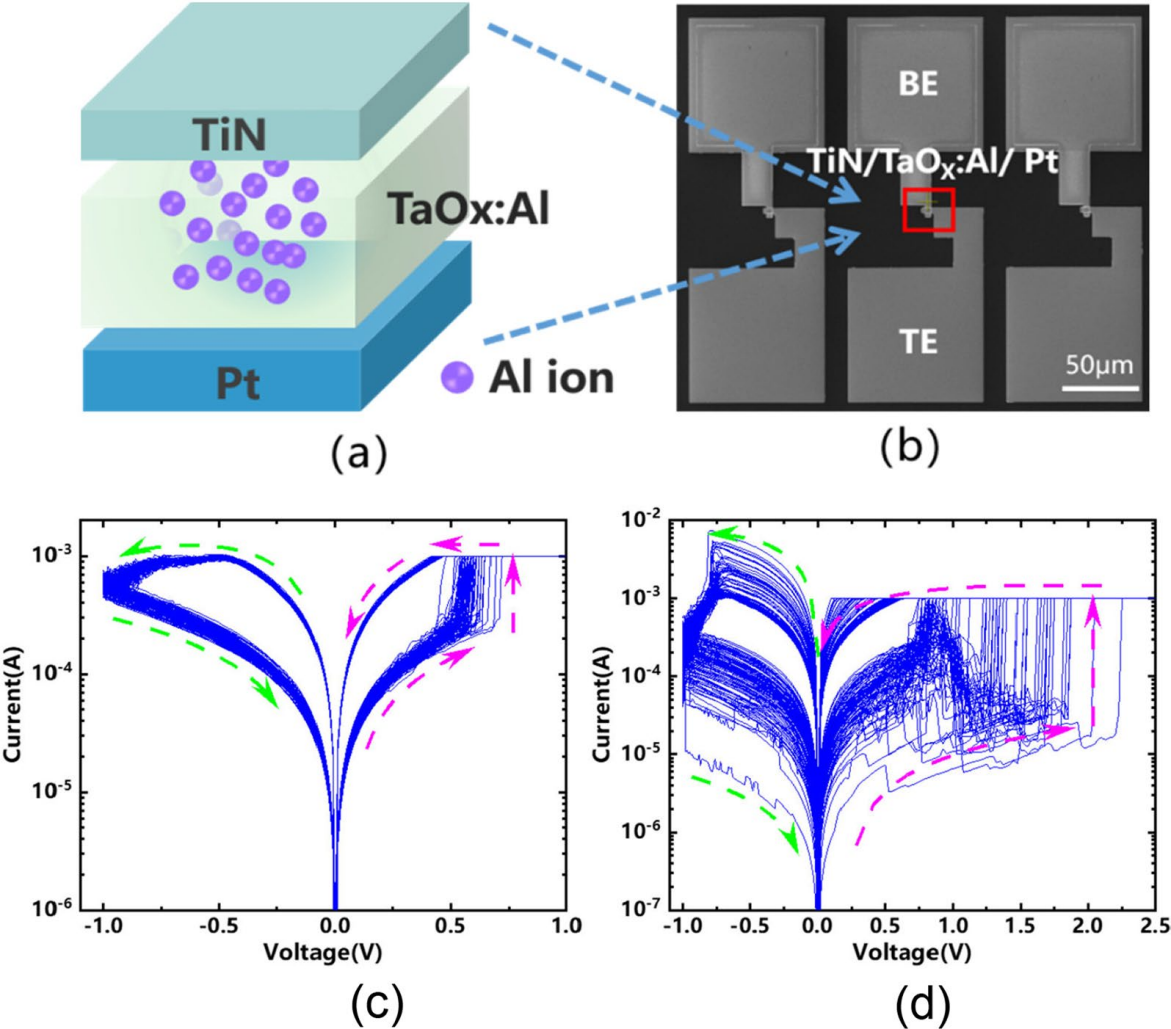
**a** Structure diagram of Pt/Ta$O_{x}$:Al/TiN RRAM **b** SEM image Al doped of Ta$O_{x}$ -based RRAM. **c** I–V curve of Pt/Ta$O_{x}$:Al/TiN RRAM **d** I–V curve of Pt/Ta$O_{x}$/TiN RRAM. Reproduced with permission from [100]

In RRAMs, another significant figure of merit for device manufacturing on a larger scale is the uniformity. The uniformity of operating voltage, speed, resistance in HRS and LRS and some other parameters will determine how easily RRAMs can be accommodated into a large scale and multifunctional circuit. The switching voltages as well as resistances of both the HRS and LRS are among the parameters which exhibit a high degree of variation. In RRAMs, variations of resistance switching are observed due to temporal fluctuations (cycle-to-cycle) and spatial fluctuations (device-to-device). Cycle-to-cycle and device-to-device variability is a major hindrance for information storage in RRAM devices [84]. Cycle-to-cycle variations in RRAM can be attributed to various factors including randomness of ion migration, gradual changing of morphology of a filament or switching interface, current overshoot, and so on. The degradation in performance of RRAM is also observed due to the device-to-device nonuniformity and its origin is attributed to the nonuniformities in the fabrication process such as the thickness of the switching film, etching damages and surface roughness of the electrodes. To improve the uniformity of RRAM, various methods have been explored. One of the methods utilizes the concept of inserting nanocrystal seeds which confine the CF formation by enhancing the local electric field effect [97, 98]. In Ti/Ti$O_{2-x}$/Au-based RRAM [99], enhanced uniformity characteristics are reported due to the induction of the platinum (Pt) nanocrystals within the resistive switching layer. The Pt nanocrystals limit the switching effect into regions with high oxygen vacancy generation probability, thus resulting in improved uniformity of the device. In another approach, Qin et al. [100] reported the improvement in uniformity of TaOx-based RRAM by local doping of Al ions. Compared with a device without doping, the device with locally doped Al ions exhibited excellent uniformity characteristics with tighter distribution of operating voltage and resistance states. Figure 13a depicts the device structure of the Pt/Ta$O_{x}$:Al/TiN RRAM and the SEM image of the device with Al ion doping is shown in Fig. 13b. The current voltage (I–V) curves of the Ta$O_{x}$ -based RRAM devices with locally doped Al ions and without Al doped device under 100 consecutive cycles are depicted in Fig. 13c, d, respectively. From the I–V curves of the RRAM devices, it is observed that the Al ion-based device shows excellent uniformity compared to the undoped RRAM device.

### Multilevel cell operation

**Fig. 14:**
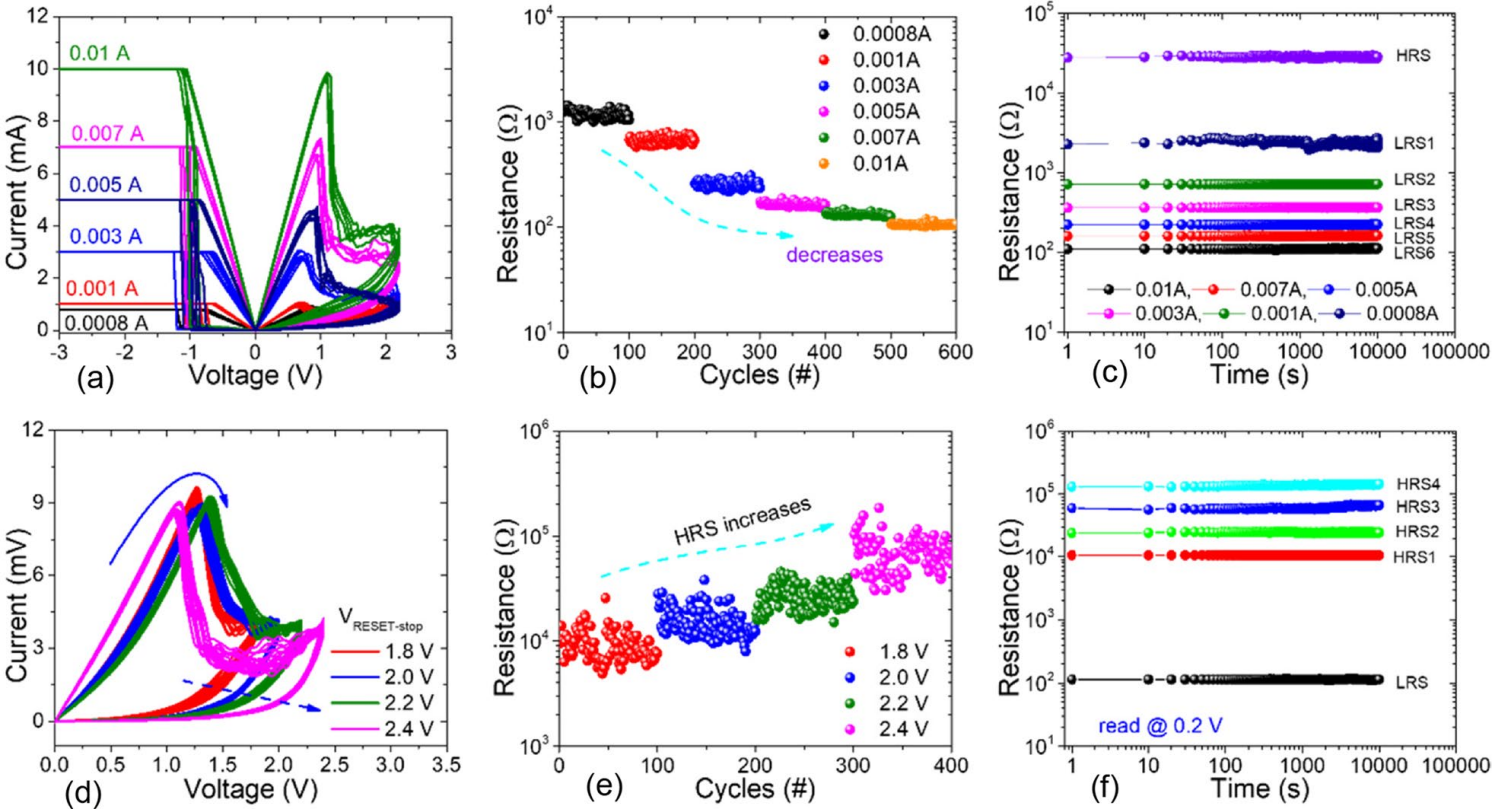
Multilevel switching characteristics of Pt/$Al_{2}O_{3}$/$HfO_{2}$/HfAlOx/TiN RRAM. **a** I–V curves with varying compliance currents. **b** DC endurance performance under varying currents. **c** State retention characteristics for 10$^{4}$s with varying $I_{cc}$ (6 LRS: 1 HRS). **d** I–V curves with varying reset voltages. **e** DC endurance characteristics at various reset voltages. **f** State retention characteristics for 10$^{4}$s with varying reset voltages. Reproduced with permission from [101]

To minimize operational cost, it is desired to increase the density of the memory devices which in turn reduces the use of the Si substrate area. Among the various memory characteristics, multilevel cell (MLC) is a desirable characteristic for realizing high-density memory applications as it exploits the layout area of the memory device for achieving more than one bit of data per cell [101, 102]. The MLC capability enables multibit storage and potentially increases the storage capacity for ultra-high-density memory applications [103]. In RRAMs, the MLC operation is achieved by the modulation of the resistance states [104, 105]. According to various reports existing in the literature, variability of the resistance in RRAM associated with a CF depends on the size of the conductive filament as well on the oxygen vacancy concentration [106, 107]. In RRAMs, the multilevel switching is commonly achieved by adjusting the LRS resistance by adjusting the values of the compliance current ($I_{cc}$) during the set cycle [108, 109]. Another method to achieve MLC operation in RRAM is by changing the HRS current by varying the maximum voltage during the reset ($V_{\max }$) operation [110, 111]. This approach, however, suffers from larger HRS resistance variability since that most depends on the ruptured filament length which varies from cycle to cycle. In MLC method by varying $I_{cc}$, the resistance of the LRS is dependent on the filament conductivity and its radius, thus resulting in a smaller variability.

Mohammad et al. [101] reported the multilevel characteristics of Pt/$Al_{2}O_{3}$/HfO2/HfAl$O_{x}$/TiN-based RRAM with desirable resistive switching properties such as low operating voltage (Set/Reset), high switching ratio (> 100) and multilevel retention time (10$^{4}$s). Figure 14a depicts the I–V curves with various compliance current values (0.0008A, 0.001A, 0.003A, 0.005A, 0.007A and 0.01A) used during the set process and correspondingly obtained different LRS values. The stability of the resistance states could be attributed to the fact due to the greater current limit applied stronger CF is produced due to the high density of oxygen vacancies produced. Figure 14b demonstrates the DC endurance performance under varying currents. The resistance performance of six different LRSs is evaluated by controlling the CC limit. The retention characteristics of the RRAM device for the seven resistance states (6 LRS, 1 HRS) at room temperature for over 10$^{4}$s under different current limits are shown in Fig. 14c. The observations clearly depict the good data retention performance of the device. The multilevel reset operation with various reset voltages (1.8 V, 2.0 V, 2.2 V and 2.4 V) is depicted in Fig. 14d, while the LRS is maintained constant. The endurance characteristics with different reset voltages are depicted in Fig. 14e. It is observed that for each reset voltage, the resistance value of HRS (${\text {HRS}}_{1}$, ${\text {HRS}}_{2}$, ${\text {HRS}}_{3}$ and ${\text {HRS}}_{4}$) shows stable behaviour. Lastly, multilevel resistance state retention data recorded using a 0.2 V voltage for 10$^{4}$s are shown in Fig. 14f. The retention characteristics for the five resistance states (4 HRS, 1 LRS) at room temperature demonstrate excellent performance of the device.

## Three-dimensional (3D) integration of RRAM

**Fig. 15:**
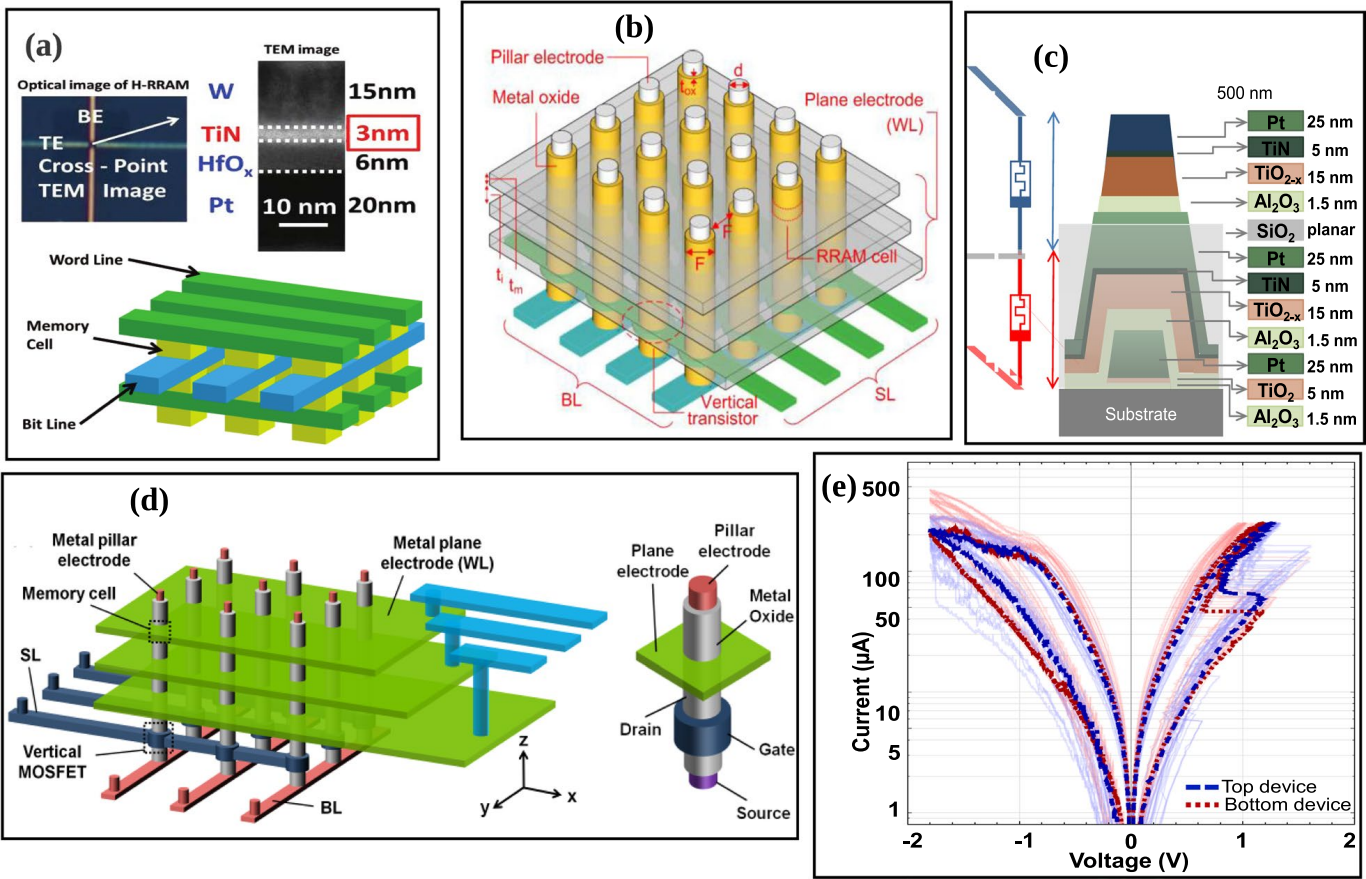
Schematic of the 3D horizontally and vertically stacked crossbar array structures. **a** Structure of the 3D horizontal RRAM array [119]. **b** Structure of the 3D vertical RRAM array [121]. **c** Circuit of a stacked memristor consisting of a shared middle electrode, shown in grey between the blue and red memristor devices [126]. **d** Structure of a 3D cross-point design showing vertical RRAM cell with vertical MOSFET [122]. **e** The plots of I–V for the red device (red dotted) and blue device (blue dotted), also shown as top and bottom device, respectively [126]

The 3D computation schematic consists of the well co-located memory elements and logic devices, and this arrangement significantly enhances the energy consumption and bandwidth access of the memory [112]. In recent times, numerous vertical RRAM architectures have been studied experimentally at a single device level and they appears to be promising [113–115]. However, to fully actualize the 3D memory system, a lot of fundamental technological issues arise and they need to be addressed, some of which include low-resistivity copper-interconnect issues with a low-k dielectric inter-layer, thermal budget incompatibility due to transistor interconnect [116, 117]. Therefore, this necessitates the assessment of the 3D RRAM system’s performance at an array level. Several modelling works have been proposed to investigate the structures of the 3D RRAM based on write/read scheme design, geometry scaling trend and the influence of parameters of the device, etc. [118–120]. This section provides the review of the progress recorded on the 3D integration of RRAM for mass storage application. The 3D horizontal RRAM array is shown in Fig. 15a. Then, the 3D vertical RRAM array is regarded as a set of vertically arranged 2D planes usually selected by the select lines (SL) as shown in Fig. 15b. The decoding is usually done by the SL, bit lines (BL) and the word lines (WL). Each vertical electrode’s edge is coupled to a WL. The pillars are joined to the BLs at the bottom of the array. SL are used to operate vertical transistors connected in series with the pillar electrodes. But the maximum height limitation for 3D arrangement for a particular etching aspect ratio (AR) can be computed when the feature size (F) is known; F can be known when the diameter of the pillar electrode (d) is added to the twice of the RRAM dielectric oxide thickness (t$_{ox}$). Also, F is regarded as the half the distance between adjacent pillar electrodes centres.

Thus, every layer is made up of a plane electrode with a thickness of ($t_{m}$) and a separation layer with a thickness of ($t_{i}$). These structural properties are also shown in Fig. 15b. Using the resistivity and these geometric factors, one can calculate the resistance of the plane and pillar interconnects. Using a bit-cost-scalable (BICS) technique, a 3D vertical RRAM architecture is used in which the memory cell is positioned between both the plane electrode and the vertical pillar as an alternative to 3D NAND flash [121]. Thanks to the straightforward one-step pillar development technique, this construction may provide high pillar density in the axis of the metal plane. Similar design was proposed by Chen et al. [122] as shown in Fig. 15d; their cell was a double-layer vertical stacked of HfOx with TiON buffer layer which assisted in the selector-less process. The good switching speed ( $\approx$ 50 ns), immune half-selected read disturbance of (>10$^9$ cycles) and good retention have shown that the introduction of the stacked double layer has greatly enhanced the switching dynamics of the cell and suggested that more layers may be introduced for better performance. Moreover, it is important to note that a large on resistance value (($R_{\rm on}$) like $\approx$ 100 k$\Omega$) may help in reducing the sneak path currents and the setting of a low WL plane resistance may enable selector-less Mb scale array [122].

The potential of artificial synapse for high integration density and their close integration with other circuit components would avoid expensive OFF-chip communications, which is essential for the low energy performance of RRAM-based artificial neural systems. The CMOL (Cmos + Molecular) circuit is an illustration of such a device [123, 124]. Multiple crossbar layers are used in 3D CMOL [125], the most sophisticated form of CMOL circuits, and this is to further improve the actual density of synapses and communication among neurons. 3D CMOL device may have a stack of multiple dielectric layer with a shared electrode as proposed in [126] and shown in Fig. 15c, e. This kind of structure possesses analog behaviour with a several distinguishable state of operation; hence, the implementation of multiple stack 3D RRAM devices compatible with CMOS design is imminent in the field of artificial neural network (ANN), analog and neuromorphic computing [126]. Moreover, Wang et al. proposed a multilevel flexible 3D memristor device, and this design has shown a multilevel data transmission and a power consumption of 4.28 aJ and 50 ns response speed [127]. However, these designs are based on 3D horizontally stacked crossbar array structures which may encounter serious issues as the stacked layer increases. These issues are mostly due to interconnection lines that link the bottom circuits [128]. Therefore, the deployment of a 3D vertically stacked crossbar array structures may be able to solve the issues faced by the horizontal structure as shown in Fig. 15d. Moreover, the main advantage of this vertically based 3D array structure is the use of only one critical photolithography step and this is considered as a reduction in fabrication cost. Thus, simplify the lithography steps when compared with the several steps during the horizontal 3D array RRAM device. However, there are two critical issues bedevilling the 3D vertical array structure; the requirement of conformal deposition and the etching of deep holes need to be done within the insulating, stacking and metal layers. Therefore, there is need to do multiple lithography and etching processes. Moreover, apart from the fabrication issues integration of the individual selectors/transistors is very challenging. But, this selector issue could be mitigated by the use of a selector-less approach [122]. Furthermore, more efforts are needed to address these challenges and make the realization of 3D RRAM crossbar array a success.

## Materials for RRAM device

RRAM has been touted as one of the next-generation memory devices, and it demonstrated a simple and new memory type with a simple sandwiched structure. The simplicity of the structure demonstrates the significance of the dielectric layer. Though, TE and BE are termed as the main conducting medium that enhances the RRAM’s electrical conductivity [26, 129]. But, the intermediate functional resistive switching layer (RS layer) provides the switching region. Therefore, the performance of the RRAM device  decisively relies on the thin film materials’ mechanism [130–135]. However, the proper understanding of these materials and mechanisms governing their operations are currently vital issues. Hence, further investigations are essential to reveal the phenomenon behind the resistive switching (RS) processes.

**Fig. 16:**
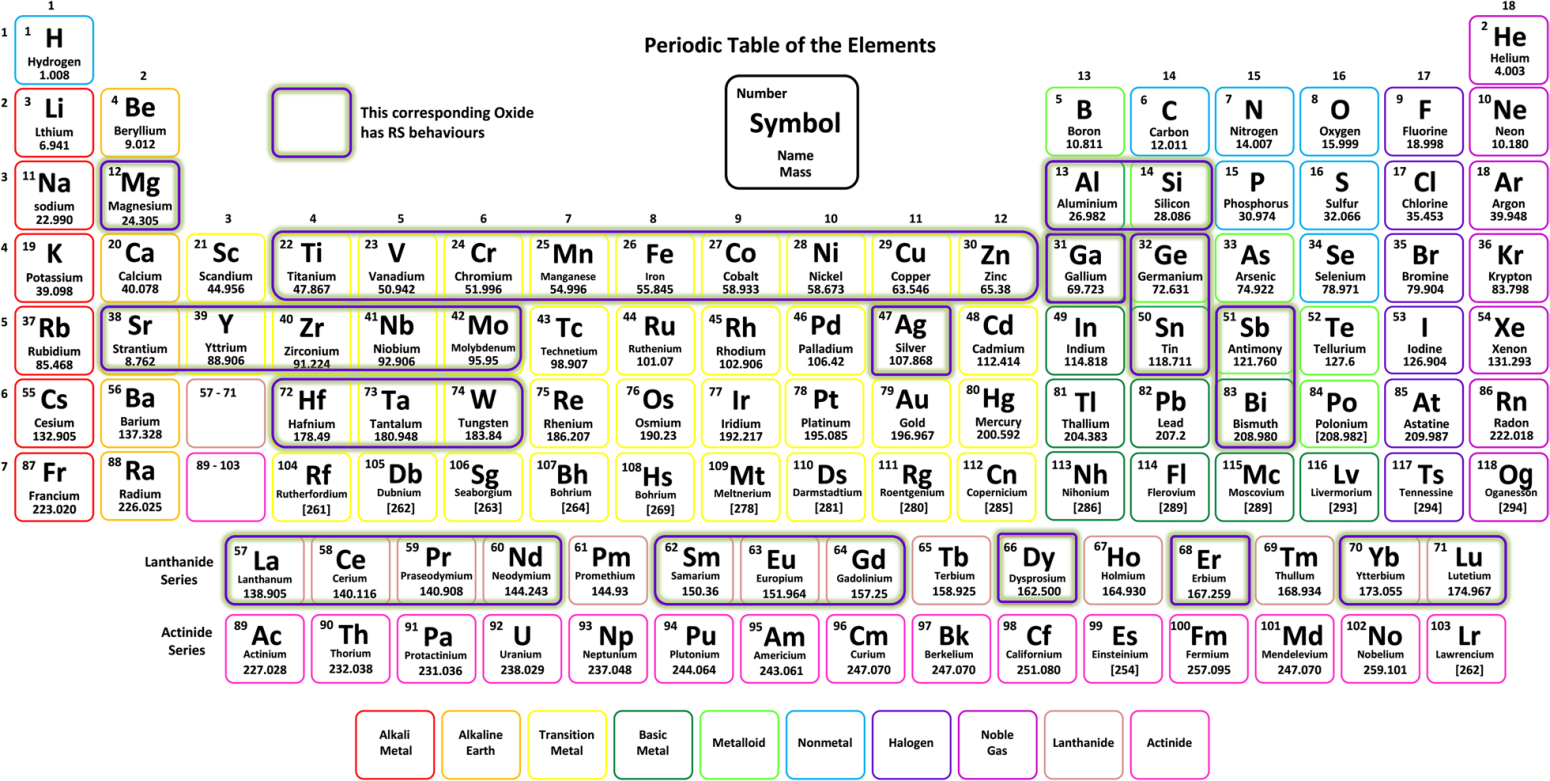
Elements that show RS behaviour highlighted in the periodic table [142, 143]

Ever since the initial interpretations of a negative differential resistance characteristics in some oxide films by T. W. Hickmott [17] in 1962, the investigation of the RS mechanism began to flourish and more materials were shown to exhibit the RS characteristics [136]. Thereafter, in 1967 Simmons et al. [137] demonstrated the RS in the Au/SiO$_{2}$/Al structure. This aids in providing both experimental and theoretical background of RRAM process. Therefore, this opened up the resistive memory potentials of some anodic oxides [138–140]. The first wave of the RS research activity had risen within 1970s to 1980s, mostly focussing on the studies and descriptions of the phenomenon behind the RS characteristics [141, 142]. The second wave of the RS research activity had been witnessed in 1990s due to the emergence of microelectronic technology processes and RS characteristics classified as the nonvolatile memory [143]. Thus far, a variety of materials are shown to exhibit the RS characteristics over decades, and most of the elements that have shown RS behaviours are shown in Fig. 16. Therefore, in this work, the RS layer is also known as the dielectric layer and classified into organic, inorganic dielectric materials and also based on the dimension of the material as classified into zero-dimensional nanomaterial (0D), one-dimensional nanomaterial (1D) and two-dimensional nanomaterial (2D). These materials have shown great potentials, but the inorganic materials outperform the organic types of material. They have excellent switching stabilities but lack in mechanical flexibility and often high cost.

### Inorganic dielectric material for RRAM

The inorganic dielectric RRAM materials encompass a large variety of binary metal oxides. These materials can be further analysed into various classes: (i) binary transition-metal oxides (e.g. ZnO [144], HfO$_{x}$ [145], TiO$_{x}$ [146], AlO$_{x}$ [147], NiO [148], CuO [149], CrO [150], MnO$_{x}$ [151], FeO$_{x}$ [152], etc.), (ii) perovskite-complex transition-metal oxides (e.g. RbPbI$_{3}$ [153], CsPbI$_{3}$ [154], CsPbBr$_{3}$ [155], etc.), (iii) chalcogenide materials (e.g. Cu$_{2}$S [156], Ag$_{2}$S [157], GeS$_{x}$ [158], etc.) and (iv) nitride materials (e.g. AIN [159], SiN [133], etc.). Furthermore, as shown in Fig. 16, the transition metals are great candidates for RRAM applications because they create numerous oxygen-deficient phases and promote the resistance switching process [130, 160]. These oxides’ hybrid is also commonly utilized as a storage medium [161, 162]. As a result of the hybrid, RRAM devices with lower threshold voltages and improved behaviour were generated compared with those using only pure metal oxide (single layer) [163]. Table 1 summarizes the switching properties of inorganic binary oxide storage materials.

Therefore, one can deduce from the details shown in Fig. 16, and the level of switching capabilities is highlighted in Table 1 that binary oxides exhibit excellent RS properties and probably the most abundant used oxides in the fabrication of RRAM devices. Additionally, binary oxide possesses good thermal stability and can easily be fabricated due to their simple structural composition.

**Table 1:** Summary of the RRAM Binary storage media and their properties

Media type	Storage Media	Switching Mode	Operation voltage ($V_{\rm SET}$, $V_{\rm RESET}$)V	ON/OFF (ratio)	Endurance (cycles)	Ref
	TaO$_{x}$	B	$\sim$2.5, $\sim$−3	$\sim$7	150	[134]
	CrO$_{x}$	B	$\sim$0.8, $\sim$−0.8	>10$^2$	6$\times$10$^4$	[151]
	MnO$_{x}$	B	$\sim$8, $\sim$2	>10$^4$	>10$^5$	[152]
	ZnO$_{x}$	B, U	$\sim$1, $\sim$−1	$\sim$10	240	[161]
	SiO$_{x}$	B	4, −2	NA	>10$^4$	[164]
	TiO$_{x}$	B, U	$\sim$2, $\sim$−1.5	2	100	[165]
	AlO$_{x}$	B	1.4, $\sim$−1.1	35	>100	[166]
	HfO$_{x}$	B	$\sim$1, $\sim$−1	$\sim$44	10$^3$	[167]
	NbO$_{x}$	B, U	$\sim$−1.09,$\sim$0.6	>10$^7$	>10$^7$	[168]
	CeO$_{x}$	B	$\sim$1.5, $\sim$−2	$\sim$10	10$^3$	[169]
	FeO$_{x}$	B	$\sim$−1.3, $\sim$1.0	800	6$\times$10$^4$	[170]
	ZrO$_{x}$	U	2.30, 0.25	10	7374	[171]
	MgO$_{x}$	B, U	$\sim$5.8, $\sim$−2.2	>10$^5$	4$\times$10$^2$	[172]
	NiO$_{x}$	U	$\sim$1, $\sim$3.5	NS	NS	[173]
	CuO$_{x}$	U	$\sim$1.2, $\sim$0.6	NS	NA	[174]
	GaO$_{x}$	B	$\sim$1.5, $\sim$−2	>10$^8$	$\sim$100	[175]
	VO$_{x}$	B	$\sim$1, $\sim$−1	$\sim$10	10$^8$	[176]
Binary oxides	WO$_{x}$	B	1, −2	>10$^2$	>100	[177]
	YbO$_{x}$	B	1.5, −1.4	NA	NS	[178]
	GdO$_{x}$	B	1.2, −1.2	>70	>10$^5$	[179]
	LuO$_{x}$	B	0.8,1.2	>10$^3$	>10$^4$	[180]
	LaO$_{x}$	U	1.3,0.3	>10$^8$	>500	[181]
	CoO$_{x}$	B	1.6,$\sim$−0.5	>100	>100	[182]
	GeO$_{x}$	B	$\sim$4,$\sim$−0.75	>10$^3$	NA	[183]
	MoO$_{x}$	B	$\sim$3,$\sim$−3	$\sim$10	>100	[184]
	SnO$_{x}$	B	$\sim$−0.2, $\sim$2	$\sim$10$^3$	$\sim$500	[185]
	SmO$_{x}$	B	$\sim$1.5,$\sim$−1.2	$\sim$2.5	10$^4$	[186]
	BiO$_{x}$	B	$\sim$1.2, $\sim$−1.2	NA	NA	[187]
	SbO$_{x}$	U	$\sim$2, $\sim$1.2	>10$^2$	>200	[188]
	YO$_{x}$	U	$\sim$2.3, $\sim$0.4	>10$^6$	NA	[189]
	AgO$_{x}$	B, U	1.8, −1.8	$\sim$10	>90	[190]
	EuO$_{x}$	B	$\sim$−1.4, $\sim$1.2	NA	<200	[191]
	PrO$_{x}$	B	$\sim$−1.8, $\sim$1.5	NA	$\sim$600	
	ErO$_{x}$	U	$\sim$3, $\sim$2	NS	>100	[192]
	DyO$_{x}$	U	$\sim$2.3, $\sim$2	NS	>110	
	NdO$_{x}$	U	$\sim$2, $\sim$1.5	NS	> 30	

$V_{\rm SET}$: SET Voltage, $V_{\rm RESET}$: RESET Voltage, B: bipolar, U: unipolar, NA: data not available and NS: not specified

Consequently, a lot of work has been done on the binary oxides with the focus especially on HfO$_{x}$, AlO$_{x}$, ZnO$_{x}$, TaO$_{x}$, CuO$_{x}$, NiO$_{x}$, SiO$_{x}$, CoO$_{x}$, TiO$_{x}$, WO$_{x}$, CeO$_{x}$, from both industrial and academic groups [37, 132]. The fabrication method of the binary oxide materials used in RRAM device often influenced the performance of the devices. Hence, discussion on the RS layer deposition techniques cannot be ignored [144]. Inorganic binary oxide storage materials are usually fabricated using various deposition techniques such radio frequency (RF) magnetron sputtering [193], thermal oxidation [194], pulsed laser deposition [195], plasma oxidation [196], electron beam evaporation [176], sol–gel method [192], chemical vapour deposition (CVD) [197] and atomic layer deposition (ALD) [198].

**Fig. 17:**
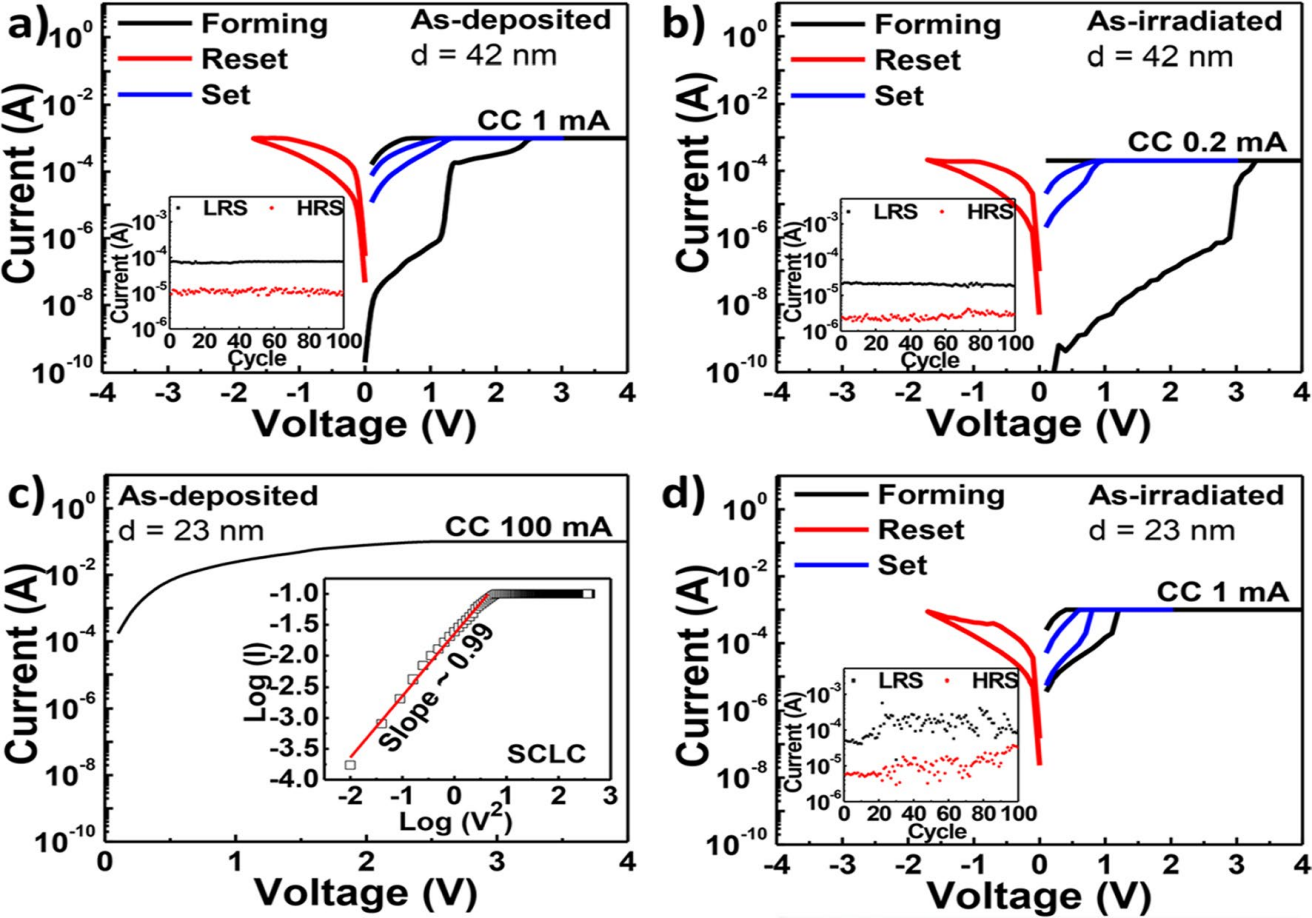
Characteristics I–V curves of Cu/ZnO/ITO device structure showing as per-deposited and as per-irradiated devices with 42- and 23-nm ZnO RS layer thickness. **a** and **b** The insets of the as per-deposited and as per-irradiated 42-nm-thick ZnO-based devices, respectively. **c** and **d** The insets of the as per-deposited and as per-irradiated 23-nm-thick ZnO-based devices, respectively. The insets illustrate the significance of the oxidation process to the RS layer of the ZnO [193]

Generally, RF magnetron sputtering method and ALD are the most frequently used and are proven to produce stable RS layers after the fabrication. Interestingly, material’s properties can be enhanced or altered by manipulating the deposition parameters during the fabrication methods. Simanjuntak et al. [193] utilize room-temperature oxidation process during the fabrication of ZnO-based RRAM device. This oxidation process provides irradiation of the neutral oxygen particles on the RS layer. Primarily, ZnO material is known as a naturally self-doped material due to the presence of abundant defects such as the zinc and oxygen interstitials; thus, ZnO-based RRAM device needs to be re-engineered for better switching characteristics [199]. From the insets shown in Fig. 17a, b, the as per-irradiated fabricated devices show a significant decrease in the compliance current (CC) for both devices thickness. The insets shown in Fig. 17c, d depict the effect of the beam-treated device on the thickness of the deposited device, the as per-deposited devices could not achieve RS behaviours because of the device thickness and high CC (leakage current), whereas the as per-irradiated device shows RS behaviours with a CC 1 mA approximately. Similarly, the effect of the deposition parameters was depicted by Kang et al. [200]. The ionic liquid (IL) gating has been an effective method for regulating device properties, and it usually effects changes in the crystalline materials using electrochemical effects as observed in TiO$_{2}$ by Tang et al. in 2017 [201].

**Fig. 18:**
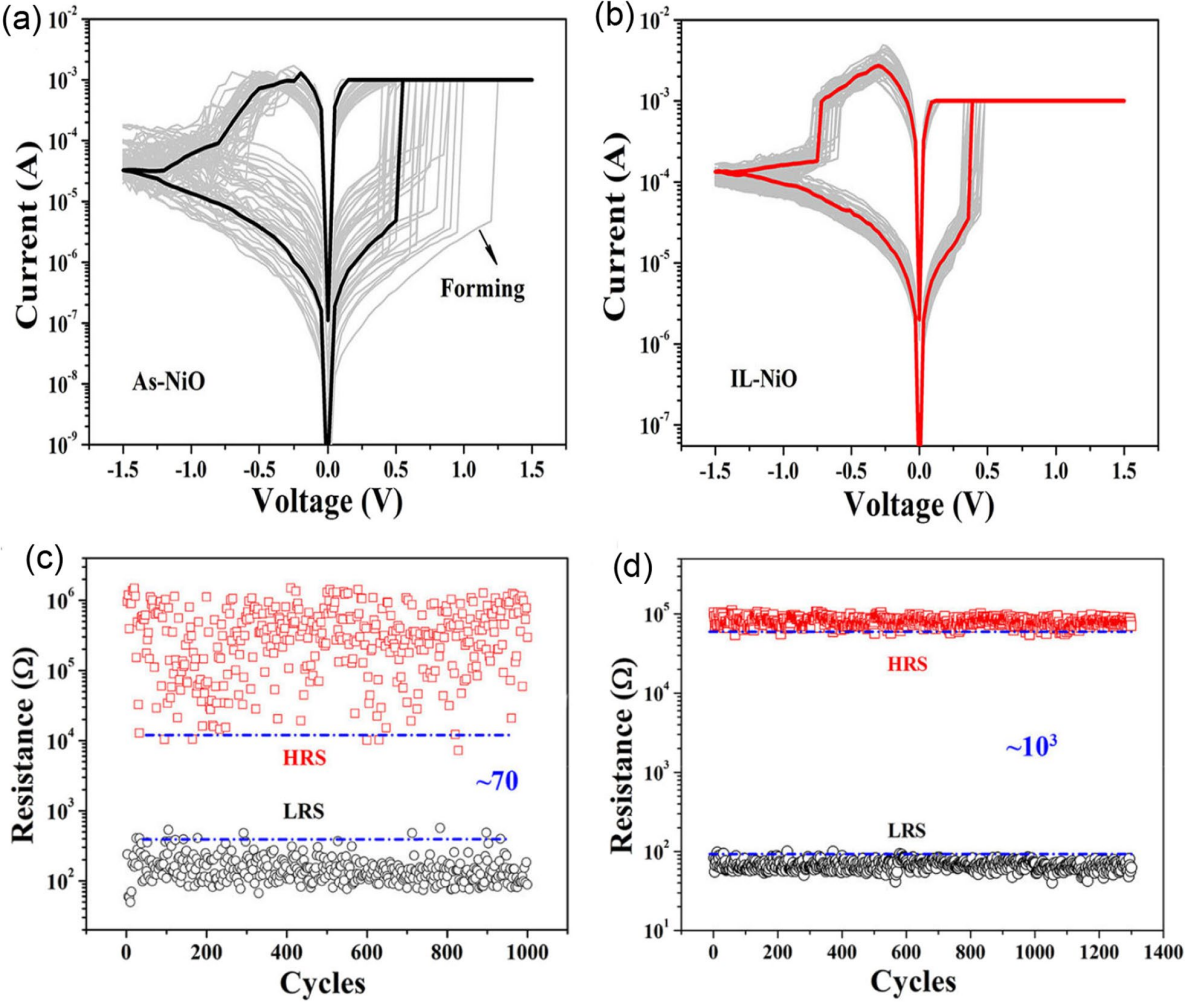
Characteristics I–V curves and endurance performance of the NiO-based RRAM device. **a** As-deposited NiO-based device I–V curves. **b** I–V curves of the IL pre-treated NiO-based RRAM device. **c** Endurance properties of the as-deposited NiO-based RRAM device. **d** Endurance properties of the IL pre-treated NiO-based RRAM device [200]

It is worth noting that the ionic liquid pre-treatment proved to be effective on the device free-forming behaviour [200], and a device treated with the ionic liquid shows enhanced RS behaviours as shown in Fig. 18a. However, the IL pre-treated device’s good performance is attributed to the formed Vo in the film and the creation of the NiO-rich layers within the edge of the NiO/Pt region. Thus, the device possesses a forming-free behaviours as shown in Fig. 18b. Moreover, the endurance performance of the device is also shown in Fig. 18c, d. From the insets, the IL pre-treated IL device characteristics shown in Fig. 18d exhibits a higher resistance ratio ($R_{\rm off}/R_{\rm on}$) of 10$^{3}$ unlike the as-deposited device with approximately 70 resistance ratio. Moreover, other post fabrication treatments on RRAM materials were witnessed recently. Post-microwave treatment (MWT) was adopted during the fabrication of SnO$_{2}$-based RRAM device by Yun et al. [202], and the SnO$_{2}$-based RRAM-treated device shows low operational current when 10 mTorr working pressure (WP) was used. Though various electrode materials were tested, the treated process (MWT and MP mechanism) proved to be effective on the developed devices [202].

**Fig. 19:**
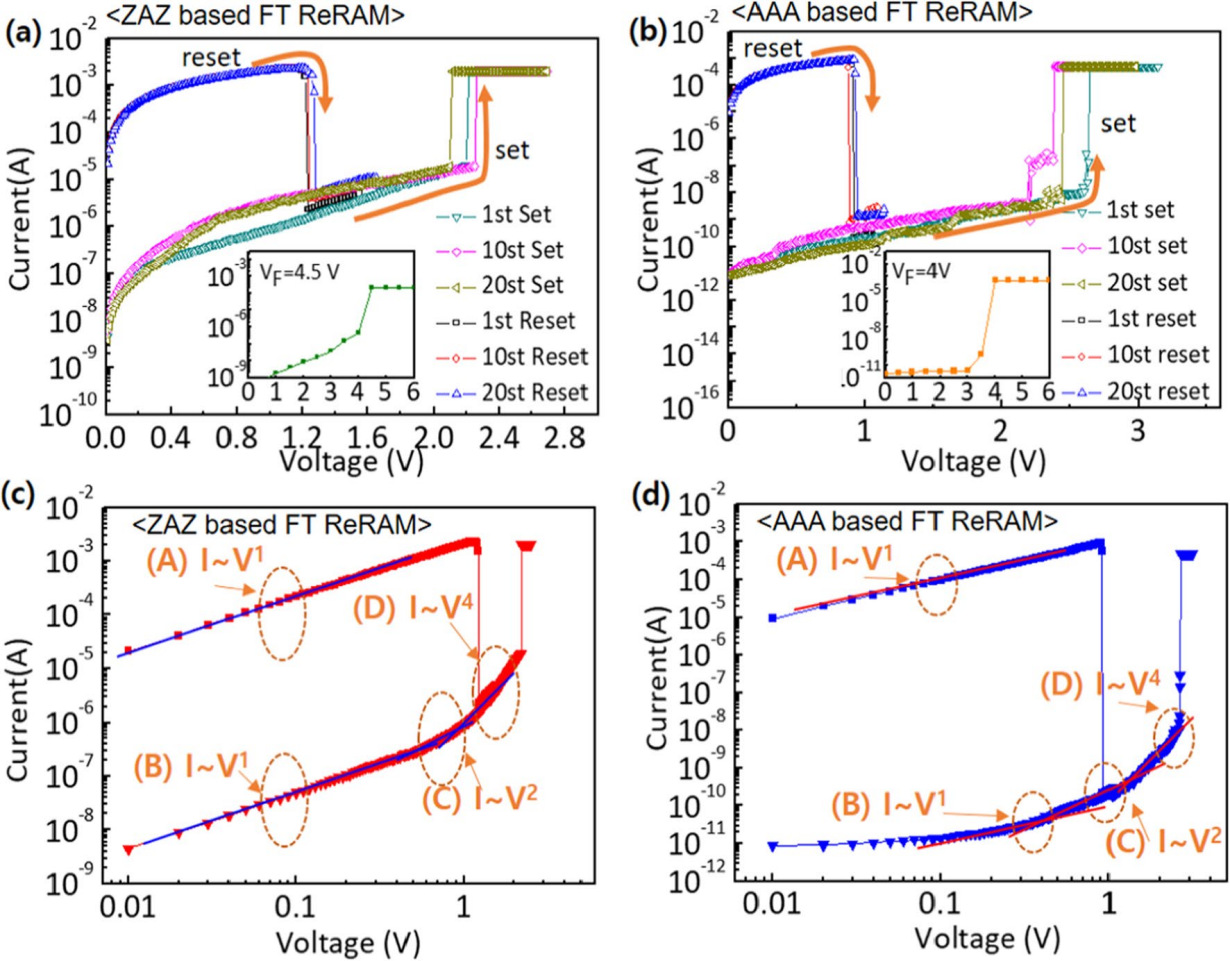
I–V current characteristics showing the forming process of **a** ZnO/Ag/ZnO device (ZAZ) and **b** crossly stacked layers of Al$_{2}$O$_{3}$/Ag/Al$_{2}$O$_{3}$ (AAA). The logarithmic plots of the I–V characteristics of **c** ZAZ device **d** AAA stacked layers device. These unipolar I–V characteristics of the devices show that flexibility or transparency does not hinder the operational characteristics of WMC devices [208]

Recently, interest has been shown on TaO$_{x}$, HfO$_{x}$ for their superior operation speed and good endurance cycles [203, 204] and capabilities for neuromorphic computing application [26]. Also, ZnO$_{x}$ and WO$_{x}$ have been studied and shown to possess multifunctional capabilities [37, 205]. The ability of these RS materials to be fabricated on wide morphological settings makes them multifunctional materials. Recently, wearable and computing memory devices (WMC) have witnessed tremendous interest due to their diverse applications. Therefore, attention would now be focused on RS materials such as ZnO and WO due to their vast potentials. WMC devices have reported use in a variety of fields, including clothing and smart watches, stretchable, wearable, transparent and soft electronics, etc. [206, 207]. For instance, the development of invisible and flexible ZnO-based memory by Lee et al. [208] could be a plus to the realization of the future WMC devices. It is worth noting that being flexible or transparent does not hinder the performance and operational stability of the WMC devices [206]. The insets shown in Fig. 19 depict the operational characteristics of a flexible transparent memory device, and it shows that the flexibility or transparency does not obstruct WMC’s operational performance.

### Other dielectric material for RRAM

Furthermore, other materials have been explored and had shown to be promising storage materials. Many dielectric materials such as perovskite oxides, nitrides, perovskites, chalcogenides, organic materials, hybrid oxides and two-dimensional (2D) material like graphene were explored as given in Table 2.

**Table 2:** Summary of the switching properties of some other dielectric materials

Media type	Storage Media	Switching Mode	Operation voltage ($V_{\rm SET}$, $V_{\rm RESET}$)V	ON/OFF (ratio)	Endurance (cycles)	Refs.
Perovskite Oxides	BiFeO	B	$\sim$4, $\sim$−3	>10	NS	[209]
	SrTiO$_{3}$	B, U	$\sim$3, $\sim$−3	$\sim$10$^6$	>10$^4$	[210]
	PCMO	B, U	1.5, −1.5	10$^3$	$\sim$120	[211]
	BaTiO$_{3}$	B	2.2, −3	$\sim$58	200	[212]
	LaAlO$_{3}$	B	2, −3	>10$^4$	>10$^2$	[213]
Nitrides	SiN	B	$\sim$6, $\sim$−3.8	100	NA	[133]
	AIN	B	$\sim$1,$\sim$−1	>10$^3$	NA	[160]
Organometal Halide Perovskites	CsPbI$_{3}$	B	0.18, −0.1	10$^6$	>300	[214]
	RbPbBr$_{3}$	B	−2.4, 2.5	10$^5$	>2000	[215]
	CsPbBr$_{3}$	B	$\sim$0.7, $\sim$−0.9	10$^6$	>10$^3$	[216]
	CH$_{3}$NH$_{3}$PbI$_{3}$	B	0.7, $\sim$−0.5	10	>400	[217]
	CH$_{3}$NH$_{3}{-}$PbBr$_{3}$	B	3, −4	10$^3$	>240	[218]
	RbPbI$_{3-x}$Cl$_{x}$	B	$\sim$2.14, $\sim$−2.14	NS	>800	[154]
Chalcogenides	GeS$_{x}$	B	$\sim$0.2, $\sim$−0.75	NA	10$^3$	[159]
	CU$_{2}$S	B	NS	>10$^5$	>10$^5$	[157]
	Ag$_{2}$S	B	$\sim$1, $\sim$−1	$\sim$10$^5$	100	[158]
Organic materials	GO	B	$\sim$2, $\sim$−1.5	NS	$\sim$10$^3$	[219]
	Indole derivatives	B	$\sim$−2.45, 2.58	$\sim$10$^6$	NA	[220]
	Sericin	B	2.5, −1	10$^6$	NA	[221]
	Cellulose-gelatine	B	1.33, −1.42	NA	500	[222]
	Egg albumen	B	0.6, −0.7	$\sim$100	10$^4$	[223]
	Pectin	B	3.3, −4.5	450	100	[224]
	Parylene	B	$\sim$2, −4	$\sim$10$^4$	>10$^3$	[225]
	Leaves	B	1.5,$\sim$−1.5	$\sim$30	NA	[226]
	Keratin (hair)	B	$\sim$6,$\sim$−6	$\sim$180	NA	[227]
2D Materials	Graphene	B	$\sim$0.5, $\sim$−0.3	$\sim$60	>100	[228]
	MoS$_{2}$	B	0.18, −0.08	10$^7$	>10$^3$	[229]
	h-BN	B	0.7, −0.5	NS	$\sim$50	[230]
	WS$_{2}$	B	1.62, −1.45	$\sim$10$^3$	$\sim$10$^4$	[231]
	Black phosphorus	B	${-}$0.89, ${+}$1.95	>10$^7$	600	[232]
Hybrid Oxide	AlO$_{x}$/ZnO$_{x}$	B	$\sim$−1.0, $\sim$−1.0	$\sim$10$^2$	>10$^4$	[164]
	ZnO$_{x}$/BiFeO$_{x}$	B	$\sim$2.0, $\sim$−2.0	$\sim$10	NA	[165]
	ZnO$_{x}$/GO	B	0.23, −0.2	10$^3$	$\sim$10$^3$	[166]
	PVP:GO/HfO$_{x}$	B	0.6, −1.46	10$^5$	>800	[233]
	CsPbBr$_{3}$/Cu$_{2}$ONdO	B	$\sim$2, $\sim$−2	10$^3$	$\sim$10$^3$	[234]

$V_{\rm SET}$: SET Voltage, $V_{\rm RESET}$: RESET voltage, B: bipolar, NA: data not available and NS: not specified

As thin films in RS memory devices, various perovskite oxide materials, such as BiFeO, SrTiO$_{3}$, BaTiO$_{3}$, Pr$_{x}$Ca$_{1-x}$MnO$_{3}$ (PCMO) and LaAlO$_{3}$ , have been extensively studied. Even though perovskite oxides have shown some promising characteristics in RRAM application, they have some common manufacturing limitations that hinder their adoption in new applications like the processing in high-temperature domain, rigid and brittle ceramic films (not flexible) and their complex constituents make it difficult to control their stoichiometry. Therefore, attention has begun to be shifted to organometal halide perovskite materials (OHP) that have shown good performance in the RRAM devices [217, 218]. These kinds of perovskite material consist of three fundamental constituents (ABX$_{3}$): A is the organic cation (CH$_{3}$NH$_{3}$), B is the metal cation and finally X is the halide anion of either iodine (I), bromine (Br) or chlorine (Cl).

However, due to the organic groups in the OHP materials, they are associated with an inherent thermal and photostability constraints due to the interaction with moisture under ambient conditions. On the other hand, by substituting organic cations with inorganic cations such as Cs, the stability of OHPs can be enhanced while retaining their structural and electrical properties [235]. Also, the OHP material can have moisture and air stability when octylammonium halide is used as the capping agent [236]. The presence of a capping agent with a long alkyl chain provides the materials with enhanced moisture and air stability as well as good ease of processing from common solvents. Moreover, the OHP materials can also enhance their operational characteristics via chloride doping, and the reliability, bandgap, defect density and on/off resistance ratio (from 2 to 500) of the device increase with the increase in the chloride doping, and also, the chloride doping makes the device to be stable even after six (6) months of development [218]. Further modifications in electrical characteristics may be feasible in these materials by adjusting the organic, metal, halide and/or capping agent of the basic perovskite nanoparticles configuration for future artificial intelligence and big data era [237].

**Fig. 20:**
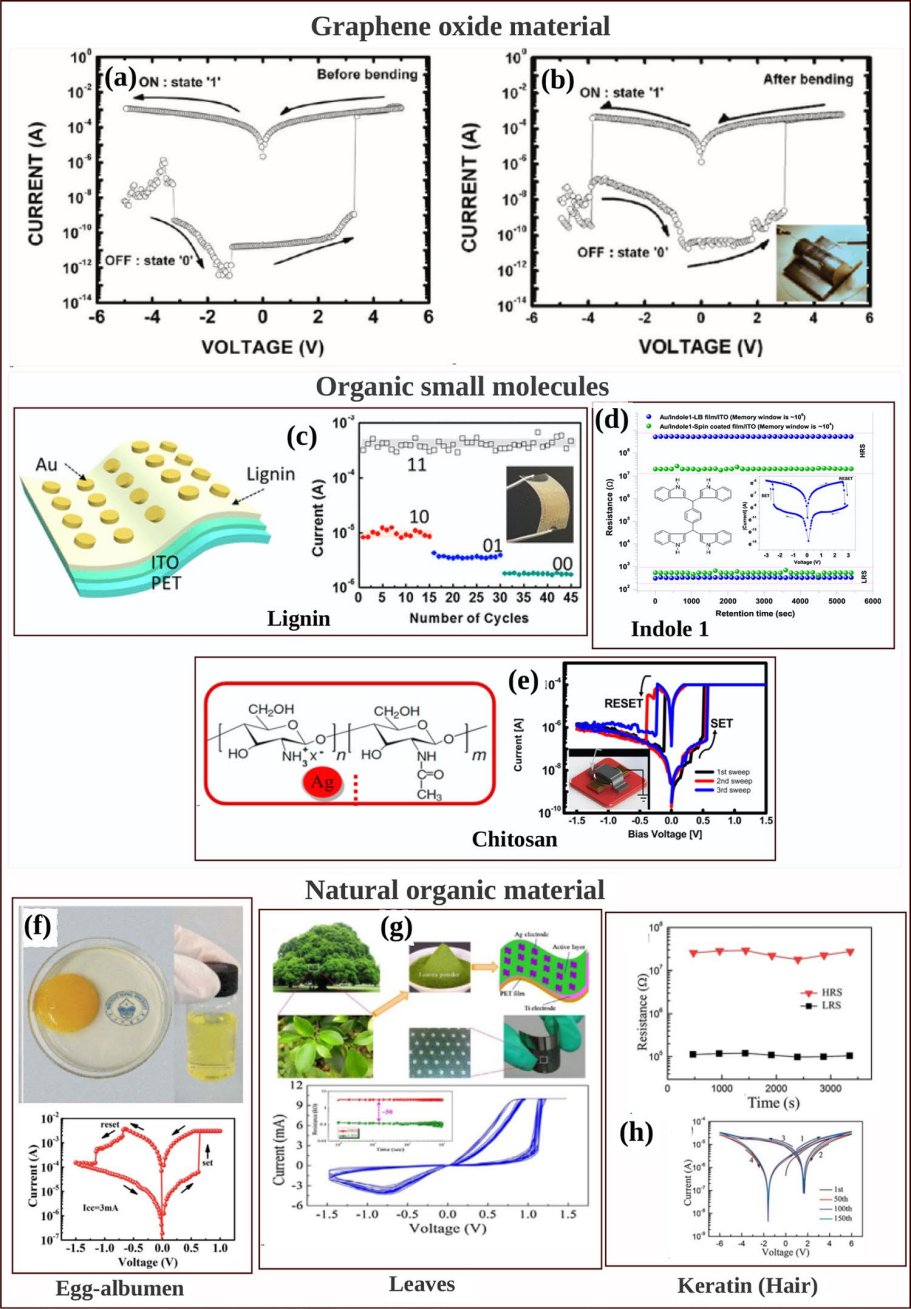
Organic materials with resistive switching. **a** Flexible graphene-based RRAM of Al/PMMA/graphene/PMMA/ITO/PET before bending. **b** The Al/PMMA/graphene/PMMA/ITO/PET after bending surface curvature radius (R) of 10 mm. **c** The structure of Au/lignin/ITO RRAM device and multilevel resistance characteristics of the device. **d** The chemical structure of the indole 1 molecule, the I–V curves and the retention time of the Au/Indole1/ITO RRAM device. Blue depicts the LB film and green represents the spin-coated film. **e** The Ag-doped chitosan device unit and the I–V characteristics of the chitosan-based RRAM device with AgNO$_{3}$ concentration. **f** Chicken egg, extracted egg albumen and typical electrical characteristics curves of the Ag/egg albumen/ITO/PET. **g** The preparation process of the plant leaves-based RRAM device, the retention time and the I–V characteristics curve of the developed Ag/Leaves/Ti/PET device. **h** The I–V resistive characteristics curves of Ag/keratin/ITO RRAM device and good retention time. **a**, **b** Reprinted with permission [240] Copyright Jul 2010, American Chemical Society. **c** Reprinted with permission [241] Copyright Feb 2017, American Chemical Society. **d** Reprinted with permission [220] Copyright Apr 2021, American Chemical Society. **e** Reprinted with permission [242] Copyright Jan 2015, American Chemical Society. **f** Reproduced with permission [223] Copyright 2017, Royal Society of Chemistry. **g** Reproduced with permission [226] Copyright 2018, Elsevier Inc. **h** Reproduced with permission [227] Copyright 2019, Royal Society of Chemistry

Another form of RRAM materials that are attracting huge interest is organic-based materials, and these materials are economical, easily fabricated, stacked and scaled. Among these organic materials, GO and rGO have been extensively studied and proven to be promising candidate for RRAM applications [238]. Graphene as one of the most explored organic materials has been used in electronic characteristics tuning and to provide flexibility in RRAM devices [239]. Son et al. developed a graphene-based flexible RRAM device [240], and the flexibility of the device does not hinder its operational characteristics even after 1.5 x 10$^{5}$ times of being bent as shown in Fig. 20a, b.

Moreover, more organic materials have been explored and exhibit good memory characteristics. Lignin is classified as a complex organic polymers usually found in a support tissues of most plant. The memory potentials of lignin were shown in Au/lignin/ITO RRAM structure [241]. The developed lignin-based RRAM device shows multibit data capabilities as shown in Fig. 20c and good mechanical strength apart from good electrical characteristics.

Remarkably, self-standing films of Indole derivatives mixed with clay particles were found to demonstrate optical switching using Langmuir–Blodgett (LB) and spin-coated techniques [220]. Thus, the electric field-driven conduction and the oxidation–reduction process are the bases behind the high device yield, good retention time and good device stability as shown in Fig. 20d. Nonvolatile memory characteristics are also reported in a chitosan-based RRAM devices developed using inexpensive solution-assisted processes techniques [242]. Chitosan is a natural solid polymer electrolyte that is biocompatible, less expensive and environmentally friendly. The developed device shows good retention time and electrical characteristics as shown in Fig. 20e. Natural biomaterials have continued to show resistive memory promising characteristics due to their flexibility and mechanical endurance. Interestingly, natural biomaterial like egg albumen has also been explored and proven to be a promising flexible RRAM material [223]. The RRAM device based on egg albumen developed using simple water dissolution techniques demonstrates good flexibility and mechanical endurance apart from the excellent electrical switching characteristics as shown in Fig. 20f.

Biomaterials like plant leaves have been gaining attention recently [224]. Leaves as a most abundant and inexpensive material hold great promise for the next memory option. Resistive memory characteristics have been reported in plant material [226], and the plant leaves-based dielectric layer was deposited on a flexible substrate. The Ag/Leaves/Ti/PET device shows good resistance ratio and stable performance at ambient temperature as shown in Fig. 20g. Furthermore, more biomaterials were reported, as keratin protein was extracted from hair. Keratin is widely known to be abundant in epithelial tissues of animal and hair. Thus, it is inexpensive and environmentally friendly substances. Resistive switching using keratin protein was reported by Guo et al. [227], and the developed keratin-based RRAM device with the structure of Ag/keratin/ITO shows good retention time, resistance ration and good I–V characteristics as shown in Fig. 20h.

However, in the long run, organic natural biomaterials have shown to be environmentally friendly, biodegradable, abundant and biocompatible. It also performs well in the RRAM device application [220–227]. Interestingly, the use of these materials may reduce the risk of shortage in the resources and harmfulness that have been witnessed in the inorganic materials counterpart [243]. In addition, the application of bioorganic materials in RRAM devices could be a good idea of material recycling.

### 2-Dimensional dielectric material for RRAM

2D materials are referred to as thin crystalline sheets with free standing layers and large lateral dimensions, and they possess intra-layer covalent bonding and inter-layer van der Waals bonding. Because of the low van der Waals energies (40–70 meV), these layers can be easily exfoliated from their parent bulk material [244]. 2D materials are expected to make a good impact in the nanoscale devices due to their high conductivity and mobility and excellent mechanical flexibility. 2D materials as the technology drivers continue to demonstrate research and technological potential, offering a broad materials system for scientific research and the development of nano electronic devices [245]. Interestingly, 2D materials characteristics can be enhanced via doping [246], electrostatic gating [247], manipulation of the deposition parameter [248], strain and manipulation of their dimension [249] and chemical intercalation [250].

Moreover, several 2D materials such as the graphene and its derivatives, hexagonal boron nitride (hBN), molybdenum ditelluride (MoTe$_{2}$), molybdenum disulphide (MoS$_{2}$), molybdenum diselenide (MoSe$_{2}$), tungsten diselenide (WSe$_{2}$), tungsten disulfide (WS$_{2}$) and black phosphorus (BP) have been applied in various RRAM devices to obtain flexibility and optical transparency [231, 232, 251–256]. Graphene was the very first 2D material to be used in electronic component systems, accompanied by several other members of the 2D family such as MXenes, hBN and transition-metal dichalcogenides (TMDs). Graphene is the renowned 2D material to date because of its mechanical and electrical properties usually needed for RRAM’s electrode and dielectric layer of the devices [252]. Because of their extendable layer thickness, these 2D materials have remarkable thermal, physical, chemical, optical and mechanical properties, making a compelling case for them to be investigated further as the active layer of RRAMs. One other essential feature of 2D-based RRAM’s materials is their ability to withstand high temperatures without affecting working memory capacity. RRAM devices based on 2D materials provide flexible and scalable memory cells with low power consumption and fast operating speeds. Such devices also have controllable switching voltages and good ON/OFF resistance switching ratios. Further descriptions and performances of 2D-based RRAM devices are given in Table 3.

**Table 3:** Summary of the switching performance of 2D-Based RRAM devices [197, 231, 256–285]

2D-Material	Device Structure	Operation Voltage ($V_{\rm SET}$, $V_{\rm RESET}$)V	ON/OFF (ratio)	Endurance (cycles)	Refs.
Graphene and its derivatives	Pt/GO/Pt	$\sim$2.5, $\sim$−0.5	10$^4$	10$^2$	[257]
	ITO/rGO/ITO	$\sim$2.0, $\sim$−2.0	NS	10$^5$	[258]
	ITO/Graphene-ZnO/ITO	$\sim$1.0, $\sim$−2.5	7000	100	[259]
	Cu/GO/Pt	1.0, −1.0	NS	NA	[260]
	Al/GOZNs/ITOPET	$\sim$2.0, $\sim$−2.0	10$^2$	10$^2$	[261]
	Al/GO-AuNPs/ITO	$\sim$1.0, $\sim$−1.0	10$^5$	10$^2$	[262]
	Ni/PMMA-GO/ITO	$\sim$1.0,$\sim$−1.0	10$^3$	10$^4$	[263]
	Au/PVP-Graphene/ITO	2.0, −3.0	5	20	[264]
MXenes	Ag/MXene/SiO2/Pt	0,2, −0.2	10$^3$	NA	[265]
	Cu/MXene/SiO2/W	$\sim$0.6, $\sim$−1.9	NS	NA	[266]
	Ag/Ti$_{3}$C$_{2}$/Pt	$\sim$3.0, $\sim$−3.0	22 $\times$10$^4$	$\sim$500	[267]
	Pt/Ti$_{3}$C$_{2}$/Pt	$\sim$3.0, $\sim$−3.0	3 $\times$10$^4$	10$^3$	
	Al/Ti$_{3}$C$_{2}$/Pt	$\sim$3.0, $\sim$−3.0	6$\times$10$^4$	10$^3$	
	Al/Ag/MXene/MXene/ITO	$\sim$1.6, $\sim$−2.0	10$^3$	NA	[268]
Hexagonal boron nitride (hBN)	Ag/hBN/ITO/PET	$\sim$0.72, $\sim$−0.37	$\sim$100	$\sim$750	[269]
	Al/Ti/TiOx/multilayer hBN/Cu	$\sim$0.6, $\sim$−0.4	$\sim$5	NS	[270]
	Ti/thick h-BN/Cu	0.7, −0.7	10$^4$	>600	[271]
	Ti/thin h-BN/ITO	0.5, −0.3	10	>180	
	Ti/MLG/thin h-BN/MLG/Au	2.3, −0.6	10$^3$	>450	
	Ti/hBN/Au	$\sim$1.5, $\sim$−1.5	$\sim$10$^3$	1200	[272]
Transition-metal dichalcogenides (TMDs)	Ag/WSe$_{2}$/Ag	0.7, −0.3	10$^3$	NA	[197]
	Al/WS$_{2}$/Pt/Ti/SiO2/Si	1.62, −1.45	$\sim$10$^3$	NS	[231]
	Al/WSe$_{2}$/Pt	$\sim$1.62, $\sim$−1.45	$\sim$10$^3$	$\sim$10$^2$	[256]
	Cu/MoS2/W2N	2.1, −2.2	10$^3$	10$^3$	[271]
	Ti/Ni/MoTe$_{2}$/Ti/Au	$\sim$1.8, $\sim$−1.8	10	10$^2$	[272]
	Al/MoS$_{2}$/ITO	2.17, −1.62	10$^2$	10$^4$	[273]
	Ag/MoS$_{2}$/ZnO/Ti	$\sim$0.75,$\sim$−0.75	2	10$^2$	[274]
	Ag/MoSe$_{2}$/Au	$\sim$2.6,$\sim$−3.0	$\sim$50	10$^2$	[275]
	Ag/MoSe$_{2}$-doped-Se/Ag	$\sim$3.0,$\sim$−3.0	10$^2$	$\sim$500	[276]
	Ag/MoSe$_{2}$/Ti	2.2, −2.6@500K	10	NA	[277]
	Ag/WS$_{2}$/Ag	$\sim$2.3,$\sim$−2.3	10$^3$	10$^2$	[278]
	Ag/WSe$_{2}$/Ag	$\sim$0.5, $\sim$−0.3	$\sim$10$^3$	90	[279]
	Ti/MoTe$_{2}$/Au	$\sim$2.3, $\sim$−1.5	$\sim$50	NS	[280]
Black Phosphorus (BP)	AI/PFCz-g-BPQDs/ITO	${-}$1.95, ${-}$0.89	1.3$\times$10$^7$	600	[232]
	(PET)/Au/BPQD-PVP/Ag	$\sim$2.8, $\sim$−1.2	6.0$\times$10$^4$	500	[281]
	Al/BP:PS/Al	1.75, −1.25	10$^2$	NA	[282]
	Ti3C$_{2}$/BP/Ti3C2	1.5, −0.5	10$^5$	NA	[283]
	ITO/BP@PS/ITO	−0.5, 2.0	100	NS	[284]

$V_{\rm SET}$: SET voltage, $V_{\rm RESET}$: RESET voltage, B: bipolar, NA: data not available and NS: not specified

**Fig. 21:**
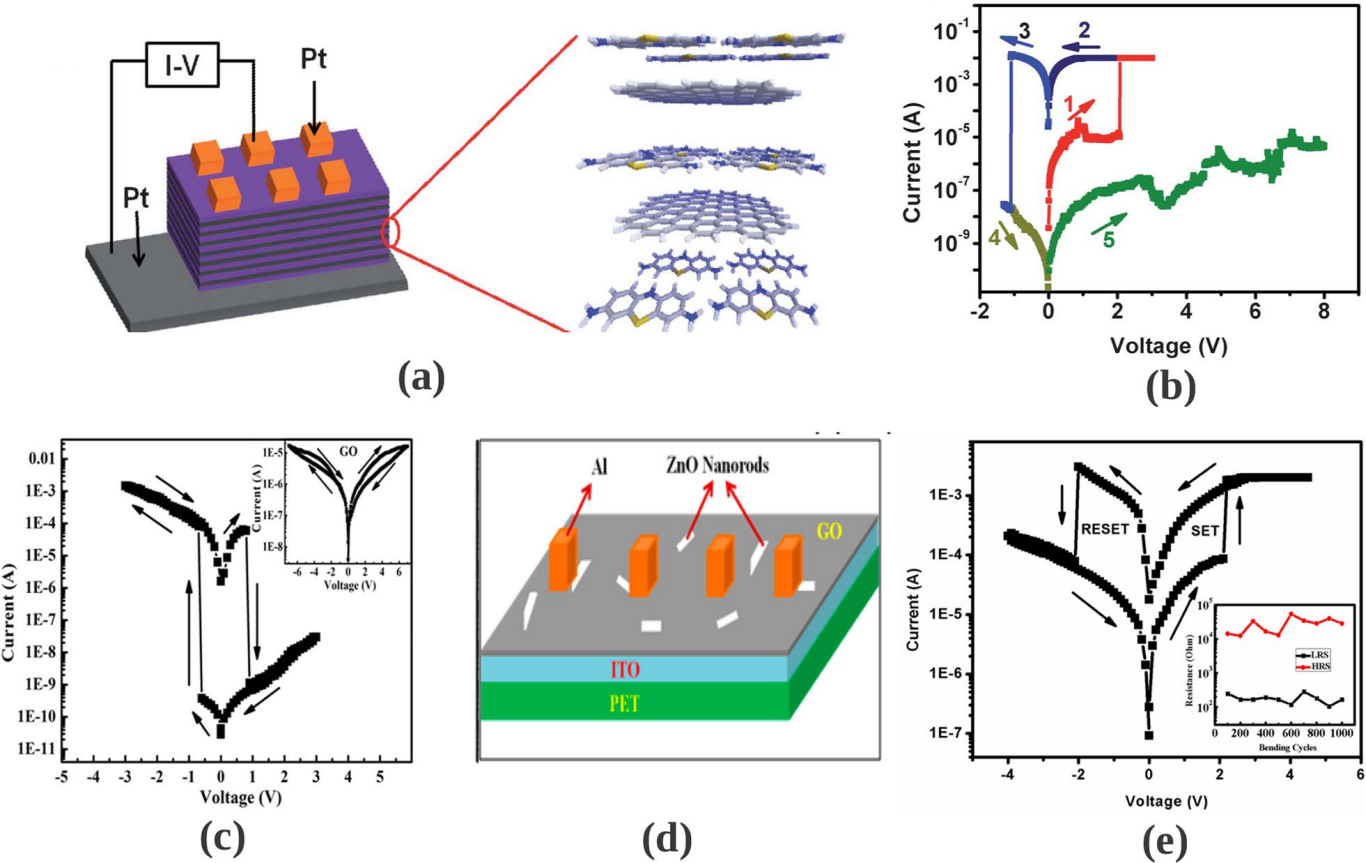
Graphene oxide 2D-based material. **a**, **b** Sketch and I–V characteristics of the Pt/rGO-th/Pt device, respectively [257]. **c** I–V characteristics of the Al/GOAu/ITO and Al/GO/ITO devices [262]. **d** Schematic orientation of Al/GOZNs/ITOPET device. **e** I–V characteristics and mechanical bending endurance of Al/GOZNs/ITOPET device [261]. The insets show various graphene oxide-based RRAM devices and their performances

Among the 2D materials, graphene is the first and most studied. Several works on graphene-based RRAM devices have been published [239]. Graphene is a transparent, flexible and inexpensive material usually applied in the form of graphene oxide (GO) [257]. Because of the presence of oxygen functionalities, graphene oxide is electrically insulating, with the added benefit of being fully atomically thin. Graphene is also used in a reduced form [258], and it can be used as an electrode to produce a desired RS characteristics [239]. However, graphene oxide embedded among dielectric layers can greatly enhance RS characteristic features such as uniformity, low operation voltage, transparency and high density [239]; incorporating GO with some other dielectric material also receives a significant attention [261–264]. The developed single-layer GO, hybrid and inserted device shows good RS characteristics as shown in Fig. 21.

**Fig. 22:**
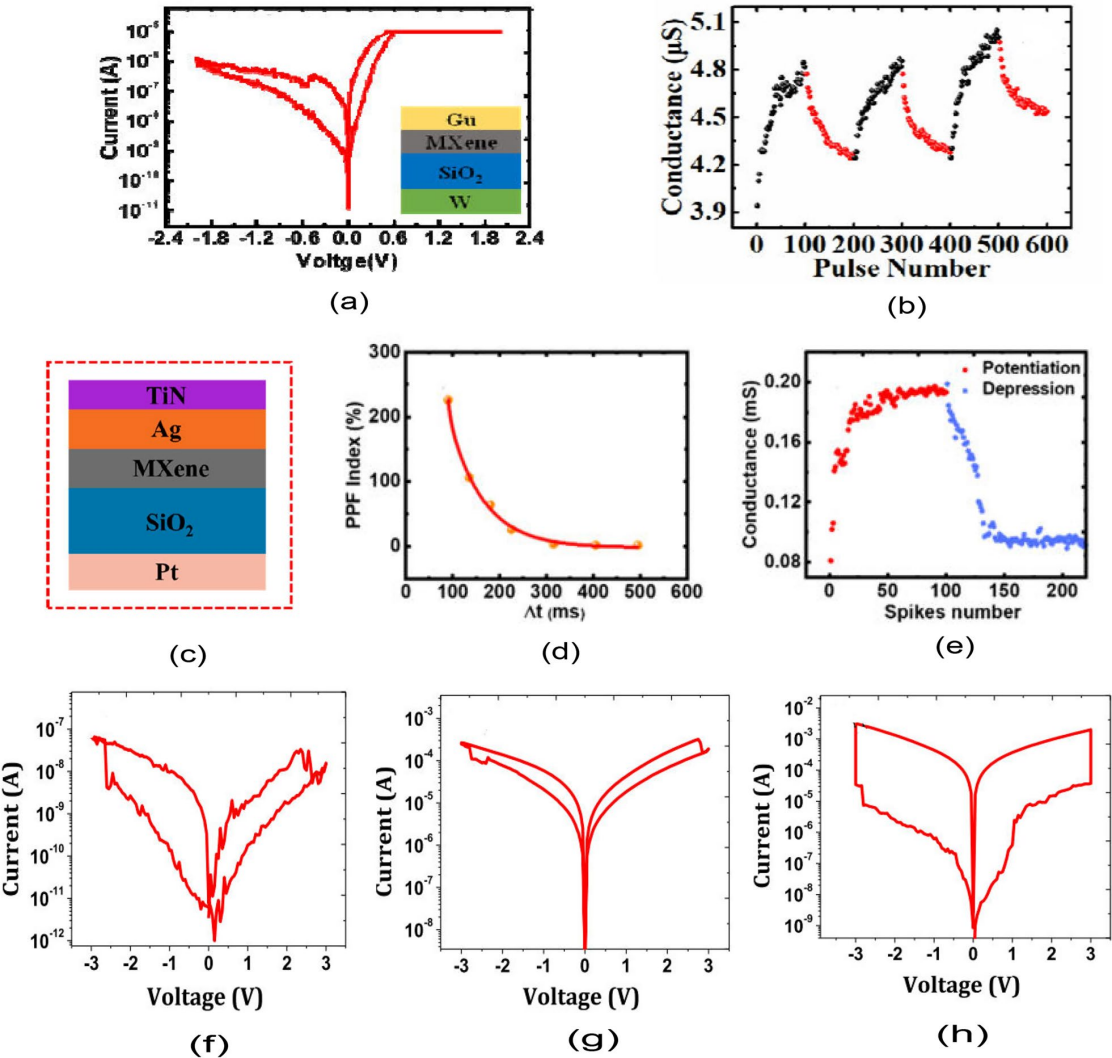
Characteristics of the MXene 2D-based material used in RRAM devices. **a**, **b** Schematic of Cu/MXene/SiO2/W device with I–V characteristics (inset) and long-term potentiation (LTP) of Cu/MXene/SiO$_{2}$/W RRAM device, respectively [266]. **c**, **d** and **e** Schematic of the TiN/Ag/MXene/SiO2/Pt, paired-pulse facilitation (PPF) index relationship and potentiation–depression characteristics of Ag/MXene/SiO$_{2}$/Pt RRAM device, respectively [265]. **f**, **g** and **h** Semi-logarithmic scales for Ag/Ti3C2/Pt, Pt/Ti3C2/Pt and Al/Ti$_{3}$C$_{2}$/Pt RRAM devices, respectively [267]

Another 2D material that receives good attention is MXenes, and it has a chemical structure of M$_{n+1}$Xn, where M and X mean the early transition metal and nitrogen or carbon, respectively. MXenes are transition-metal carbides, carbonitrides and nitrides. Because of their superior mechanical, electrical and chemical capabilities, MXenes have a substantial impact on several of applications [285]. MXene’s capability as a dielectric switching layer for RRAM is being investigated [265–268]. Because of their exceptional work properties [286], MXenes can have a broad range of ohmic contacts, which might help in improving their carrier injection ability [287]. MXene can be used as a dielectric layer or inserted/used along other dielectric materials [265, 266]. Lian et al. [265] developed RRAM device with MXene/SiO$_{2}$ as the dielectric layer, and this device shows both volatile and nonvolatile characteristics apart from good RS properties. The use of the MXene layer greatly lowers the operation voltage and provides the device with the capabilities of short-term and long-term plasticity rules; hence, this may provide a forward-looking answer for artificial synapse memory devices. Moreover, details of the RS of MXene are given in Table 3, and further explanations are shown in Fig.  22.

**Fig. 23:**
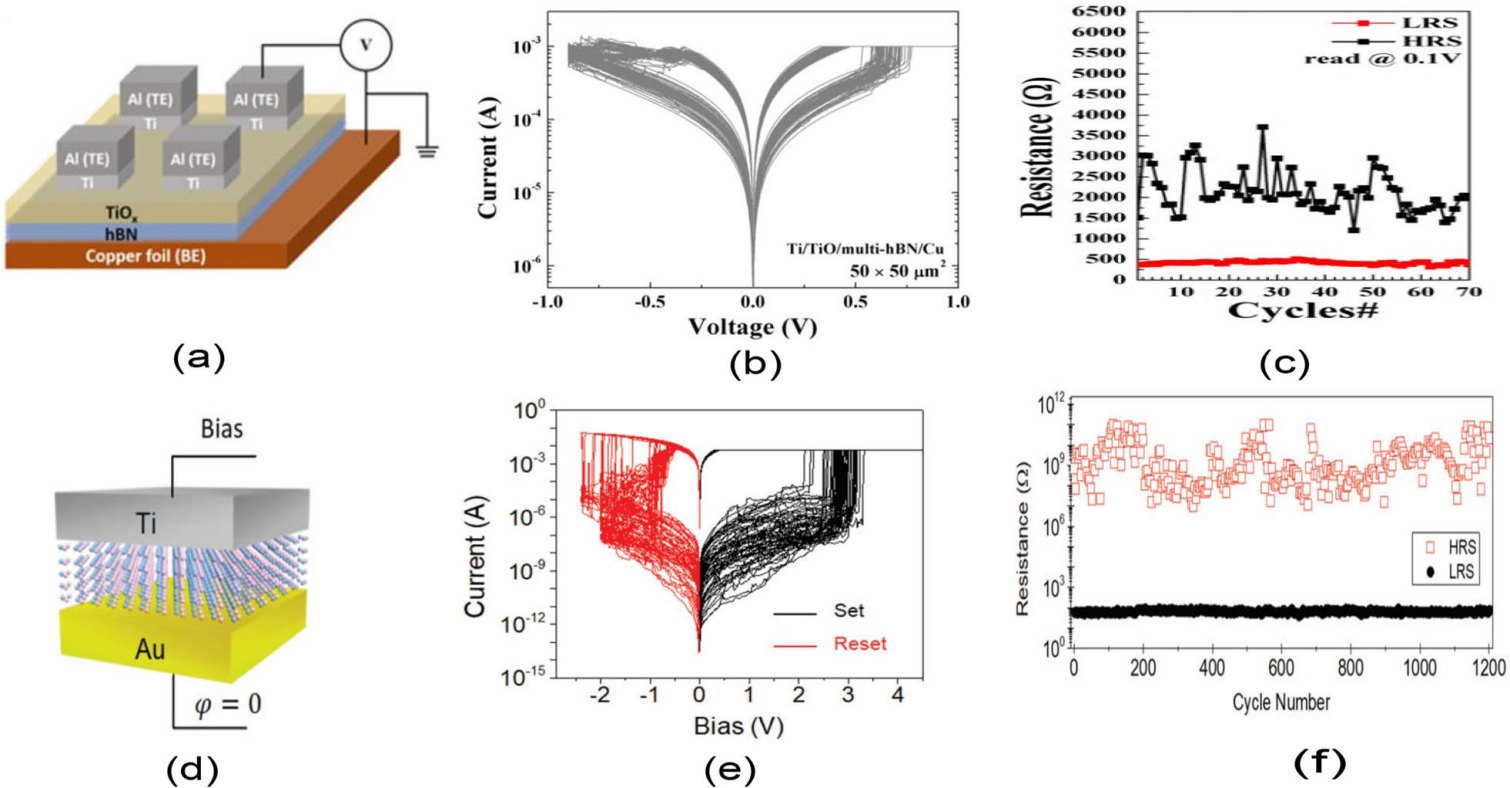
Hexagonal boron nitride 2D-based material used in RRAM devices. **a**, **b** and **c** Sketch, I–V curves and DC endurance cycles of Al/Ti/TiOx/multilayer hBN/Cu device [270]. **d**, **e** and **f** Schematic, I–V characteristics and endurance cycles of the Ti/hBN/Au device obtained at the bias of 0.05 V, respectively [272]

White graphene is a hexagonal boron nitride (hBN), and it is a unique two-dimensional material with a geometry comparable to graphene; however, a bandgap is roughly 5.9 eV [287]. RS in hBN was reported by Jain et al. [288], and the report was among the first demonstration of hBN RS capability. Their device exhibits unipolar RS characteristics but more in-depth elaborations were witnessed after this noble investigations [251] and hBN material continues to shows its potential as a dielectric layer in RRAM device [269–272]. Puglisi et al. [230] developed hBN-based RRAM device using graphene electrodes for both top and bottom, and it shows good RS characteristics. hBN-based flexible RRAM device was developed by Qian et al. [269], and this device further illustrates the capability of hBN material for the next-generation memory device. Details of the hBN materials used in RRAM devices are given in Table 3, and additional descriptions are shown in Fig. 23.

**Fig. 24:**
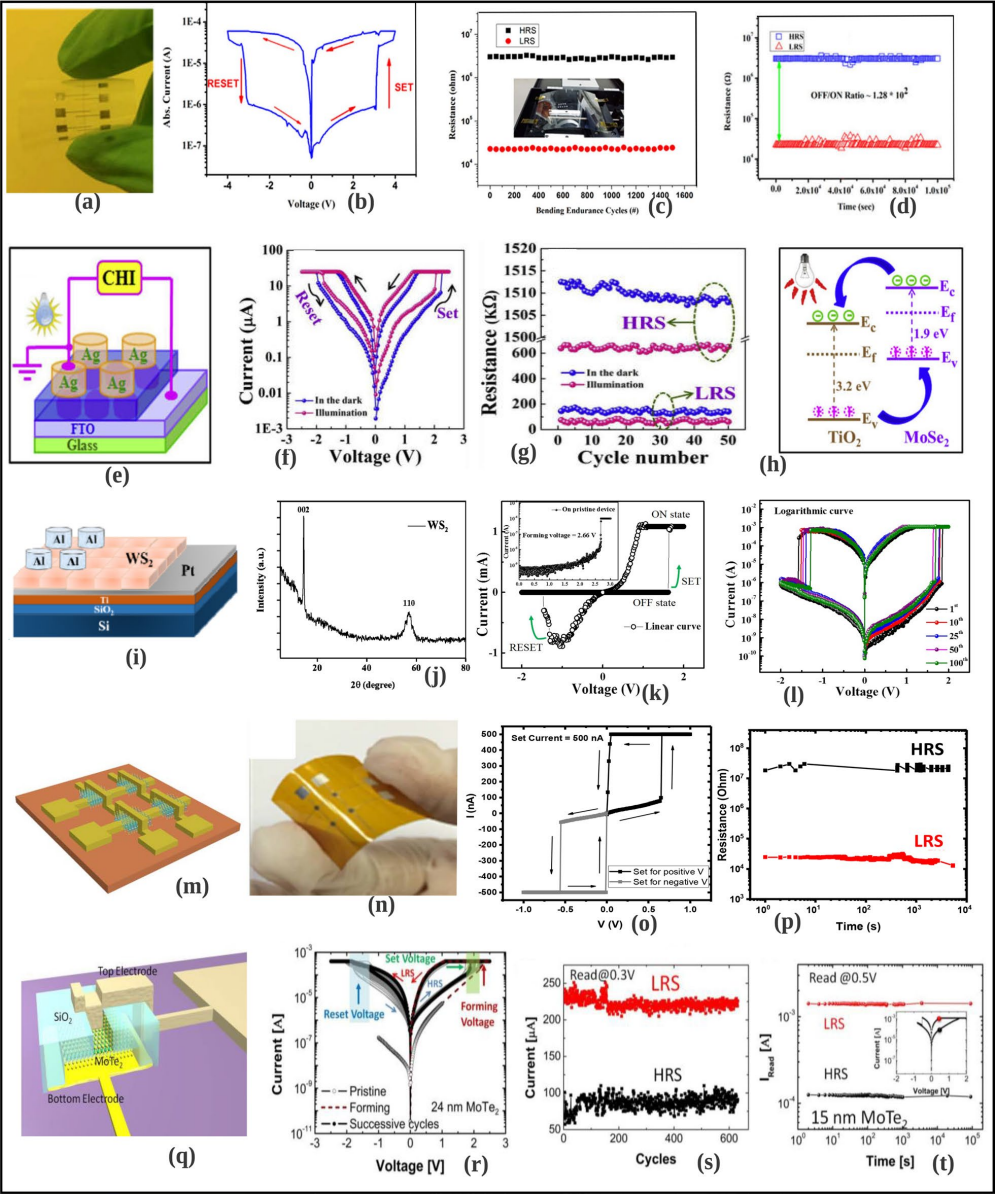
Transition-metal dichalcogenides based RRAM devices of MoS$_{2}$, MoSe$_{2}$, WS$_{2}$, WSe$_{2}$, MoTe$_{2}$ used as the dielectric layer and their operational characteristics. Details on the characteristics of MoS$_{2}$. **a** Optical image of the device developed on PET substrate. **b** Typical I–V characteristics showing the bipolar switching properties. **c** Endurance of the device showing values of 1500 bending cycles. **d** Retention properties of the device. The options (**a**)–(**d**) show various characteristics of the MoS$_{2}$-PVA-based RRAM device [291]. Details on the characteristics of MoSe$_{2}$. **e** Experimental test set-up of Ag/MoSe$_{2}$/TiO$_{2}$/FTO cell. **f** Typical I–V characteristics curve of Ag/MoSe$_{2}$/TiO$_{2}$/FTO cell. **g** Resistance cycles of the Ag/MoSe$_{2}$/TiO$_{2}$/FTO cell. **h** Electron–hole generation process of the developed Ag/MoSe$_{2}$/TiO$_{2}$/FTO cell under light illumination. The options (**e**)–(**h**) show various switching properties of MoSe$_{2}$ -based RRAM device [290]. Details on the characteristics of WS$_{2}$ material. **i** Schematic of the Al/WS$_{2}$/Pt cell. **j** Typical bipolar I–V characteristics of Al/WS$_{2}$/Pt (k) Linear current versus voltage inset of Al/WS$_{2}$/Pt cell **l** equivalent of the linear current versus voltage in logarithmic scale form showing up to 100th cycle. The options (**i**)–(**l**) show various switching properties of WS$_{2}$ -based RRAM device [256]. Details on the characteristics of the WSe$_{2}$. **m** Array Schematic of Ag/WSe$_{2}$/Ag RRAM device. **n** Image of the 2x2 array of Ag/WSe$_{2}$/Ag RRAM device. **o** DC sweep of the Ag/WSe$_{2}$/Ag RRAM device at the set current of 500 nA. **p** Retention characteristics of Ag/WSe$_{2}$/Ag RRAM cell. The options (**m**)–(**p**) show various switching properties of WSe$_{2}$ -based flexible RRAM device [256]. Details on the characteristics of MoTe$_{2}$ -based RRAM devices. **q** Schematic of a vertical TE/MoTe$_{2}$/BE cell. (r) Typical I–V characteristics of MoTe$_{2}$ -based RRAM device. **s** Current versus cycle of the MoTe$_{2}$ -based RRAM device. **t** Retention time of the 15-nm-thick MoTe$_{2}$ -based RRAM device. The options (**q**)–(**t**) show various switching properties of MoTe$_{2}$ -based RRAM device [255]

Transition-metal dichalcogenides (TMDs) are other members of the 2D material family that have shown great promising characteristics for use in RRAM devices [251]. 2D materials like MoTe$_{2}$, MoS$_{2}$, MoSe$_{2}$, WSe$_{2}$, WS$_{2}$ have been used as the dielectric layer (active layer) of the RRAM devices sandwiched between the TE and BE to form memories on rigid and flexible substrate [197, 231, 254, 255, 273–280]. The memory effect of MoS$_{2}$ was first appeared in the literature in 2012, and it was demonstrated that the bipolar RS of this developed device was due to charge carriers trapping and detrapping characteristics. Liu et al. [289] sandwiched the MoS$_{2}$ between the reduced GO and aluminium electrodes, and the layer of the MoS$_{2}$ was blended in the polyvinylpyrrolidone (PVP). Thus, it shows that 2D materials can be employed as both conducting electrodes and active materials for achieving flexibility in the future memory. Henceforth, several studies were shown on MoS$_{2}$ used as the active materials and the studies proved the capabilities of the MoS$_{2}$ for the next-generation memory devices [254, 273, 274]. More details on the characteristics of MoS$_{2}$ used in the RRAM devices are shown in Fig. 24a–d. Another class of the TMDs material is MoSe$_{2}$, and this material has been explored as the active layer of RRAM devices. A photo-controlled RS was demonstrated using the MoSe$_{2}$ inserted in TiO$_{2}$ dielectric layer, and the device has the structure of Ag/[MoSe$_{2}$/TiO$_{2}$]/FTO developed by Han et al. [290]. This shows the capability of MoSe$_{2}$ materials for use in various applications with focus on photo-controlled next-generation memory devices. Several studies show the competences of MoSe$_{2}$ materials for the next-generation memory devices [275–277], and further details are shown in Fig. 24e–h.

Another TMDs material that has been given much attention is the WS$_{2}$, and this 2D TMD material has shown promising mechanical and electrical characteristics [231]. Interestingly, the RS characteristics observed in WS$_{2}$ RRAM devices were superior to other characteristics recorded in other 2D materials [251]. The WS$_{2}$ -based 2D RRAM device was demonstrated by Das et al. [256] that exhibited good RS characteristics such as the high ON/OFF ratio and good reproducibility. Also, U. Das et al. investigate the development of WS$_{2}$ -based 2D RRAM device using chemical vapour deposition (CVD), and the CVD techniques work well as shown on the crystalline and uniformity of the film [231]. However, several other techniques were shown to blend with the deposition of the WS$_{2}$ material for RRAM devices. Moreover, good electrical and mechanical characteristics are shown in Fig. 24i–l. Another TMDs material that has shown promising characteristics is WSe$_{2}$ material. In 2019, Li et al [256] demonstrate the RS behaviours of WSe$_{2}$ material deposited on a flexible Kapton material. The device shows both volatile and nonvolatile characteristics with 2.5 h of retention capability. However, a solution-processed WSe$_{2}$ RRAM device has been investigated by Sivan et al. [279], and the employed plasma oxidation method provides a low Schottky barrier height and thus enhances the performance of the WSe$_{2}$ p-FET and enabling the device to exhibit 2.6 pJ per bit switching power. Details of the RS characteristics of the WSe$_{2}$ -based RRAM device are shown in Fig. 24m–p. Furthermore, investigation on the TMDs materials has continued to yield a lot of promising results. Another 2D material MoTe$_{2}$ has shown good RS promises. Zhang et al. [255] showed the multilevel RS characteristics of MoTe$_{2}$ exfoliated onto the Ti/Au BE using standard scotch tape methods. This 2D-based MoTe$_{2}$ RRAM device exhibits electrical field induced phase-transition characteristics unlike the conventional RRAM-based and PCM devices. It changes the state of the device between two different crystalline states; thus, it has more controllable switching mode as compared to the conventional RRAM devices that uses the ionic migration mode.

Also, this developed device shows better reliability and synaptic characteristics. As such, the capability of the MoTe$_{2}$ -based RRAM device needs to be further investigated for the realization of the next-generation memory devices. However, the characteristics of MoTe$_{2}$ -based RRAM devices are shown in Fig. 24q–t. Moreover, another electrical field induced MoTe$_{2}$ based RRAM device was investigated by Zhang et al. [280]. From their study, it shows that for the 2D material MoTe$_{2}$ to successfully function as a next-generation memory device it needs to have a tunnelling barrier of Al$_{3}$O$_{3}$ added to the material stack. Thus, this barrier will limit the amount of current moving over the cell unlike in the case of using just the MoTe$_{2}$ dielectric layer [255, 292] and provide reproducible and stable RS with a high ON/OFF current ratio of 10$^{3}$ - 10$^{4}$. Also, the device HRS state could be larger than 10T$\Omega$ which is critical to increasing the maximum RRAM array size and also reducing the static leakage power consumption of the RRAM device [280].

**Fig. 25:**
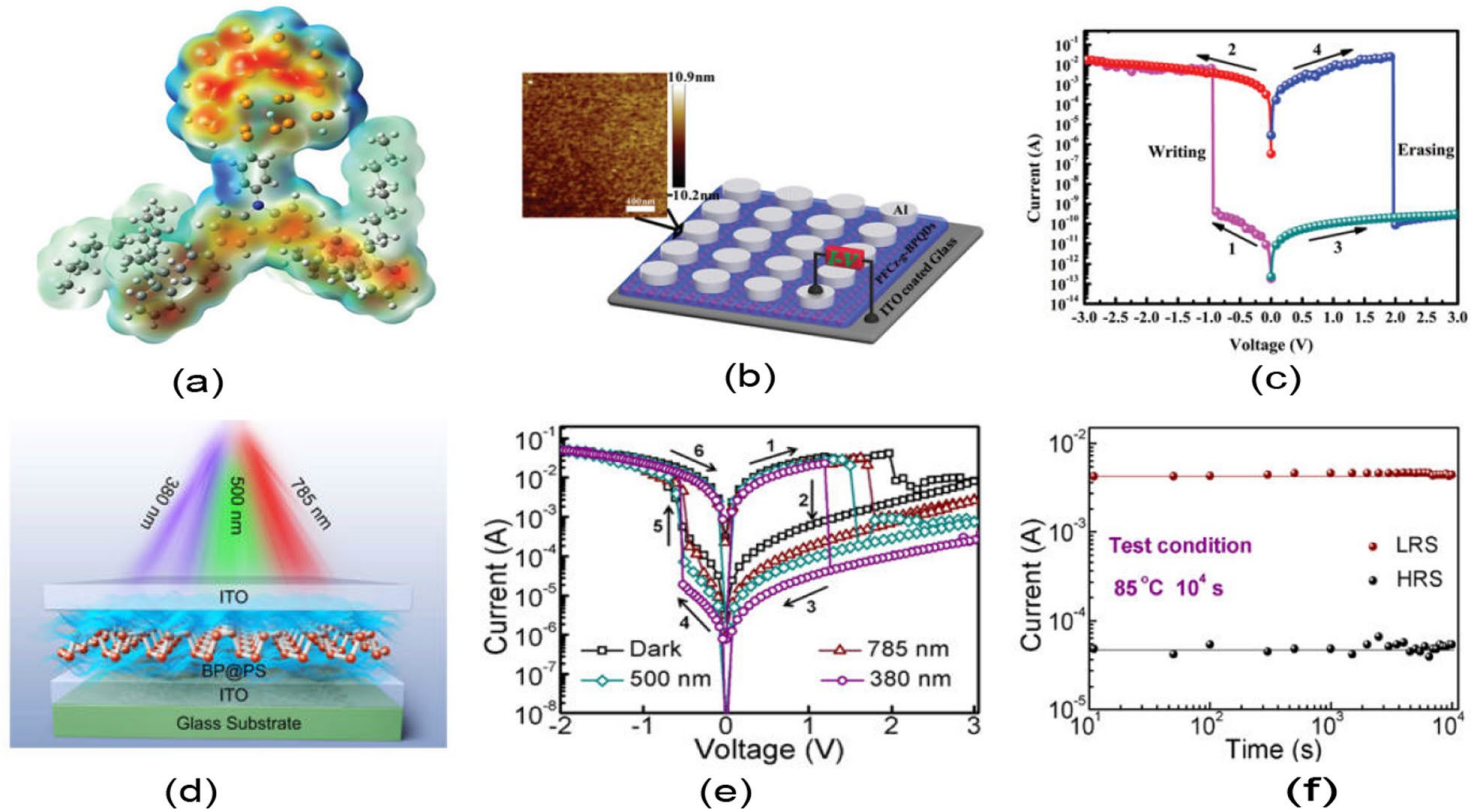
Schematic and electrical characteristics of the 2D BPQDs-based RRAM devices. **a** Surface of PFCz-g-BPQDs showing the molecular electrostatic potential (ESP). **b** Structure of AI/PFCz-gBPQDs/ITO cell with inset showing tapping mode AFM height of the cell. **c** AI/PFCz-gBPQDs/ITO I–V characteristics. **a**, **b** and **c** show characteristics of PFCz-g-BPQDs device [232]. **d** Schematic of BP@PS memristor showing light modulation **e** I–V characteristics of BP@PS memristor as modulated by different wavelengths. **f** Inset of BP@PS memristor showing its data retention. **d**, **e** and **f** show characteristics of BP@PS memristor [284]

Additionally, more 2D material emergences with great electrical and mechanical properties have been demonstrated of late and have found to be desirable for use as a switching medium in RRAM devices. Black phosphorous (BP) nanosheet is a 2D material first isolated in 2014 and does have a nonzero basic bandgap, which can be varied by strain and the layers in the stack [293]. This 2D material has the greatest thermodynamically stable form owing to its less reactivity due to an interlinked six (6)-membered ring. BP has attracted huge attention and considered a promising candidate in various applications due to its durable in-plane anisotropic physical characteristics, thickness-dependent bandgap and noticeable carrier mobility [294]. In RRAM device application, BP 2D material has shown promising characteristics [232, 281–284], and some of the studies found in the literature are given in Table III. 2D Black phosphorous quantum dots (BPQDs) have been shown to degrade under the presence of moisture and oxygen, thus termed as not stable due to oxidation [232, 295]. This effect could be mitigated via the incorporation of the BPQDs into polymer matrix or backbone, and this helps the BPQDs to form a functional materials [232]. The most reliable concept of the mitigation is to synthesize diazotated polymer (PFCz${-}$N$_2^{+}$BF$_{4}^{-}$) that usually react under aqueous conditions with the BPQDs to give the covalently modified conjugated polymer (PFCz${-}$g${-}$BPQDs) as reported by few studies [296]. Therefore, the use of one-step synthetic approach to obtain the direct covalently functionalized BPQDs with conjugated polymer shows to form a P${-}$C bond between the BPQDs and the polymer backbone. Hence, this enhances the electrical characteristics and environmental stability of the BPQDs for next-generation memories and new technology [232]. However, some of the electrical characteristics of the 2D BPQDs-based RRAM devices are demonstrated in detail in Fig 25.

## RRAM for neuromorphic computing

**Fig. 26:**
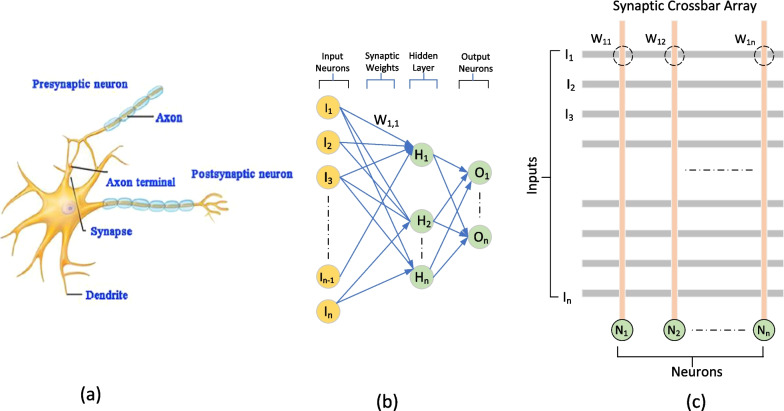
**a** Biological neural network. **b** Artificial neural network. **c** Hardware implementation of artificial neural network using crossbar [303]

The demand for next-generation technologies having enhanced performance and superior energy efficiency has been swiftly raised owing to the advancements in transistor technology towards Moore’s Law boundaries. Keeping this development in consideration, the main focus of the researchers is now on counterfeiting the activities of an amazing computational object “the human brain”. Compared with the traditional computation designs, the human brain working system has major advantages in terms of energy efficiency, consuming five orders lower energy than the modern supercomputer [297]. It is possible for the human brain to become accustomed to several situations and also to do extremely well at intricate intellectual tasks, for instance, pattern recognition. Von Neumann Bottleneck is considered to be one of the key triggers in causing this energy utilization discrepancy [298, 299]. The authorized CPUs (central processing units) and main memory regions are separated physically from each other in modern computing architectures. John von Neumann in 1945 proposed computer structure describing the design architecture for an electronic digital computer commonly referred to as Von Neumann architecture. According to this architecture, firstly the program is considered a part of data. Then, the data processed by the program and the program itself are stored with a similar memory technique. The memory addresses of both the program instruction and that of the data are physically separated locations in the same memory device; though, the width of the program instruction and that of processed data should be the same [300–302]. Moreover, the programming of these CPUs is done in such a way that these units can perform the operations in a sequential manner in which the shuttling of useful information takes place back and forth between the memory and CPU [303]. Due to this shuttling of bits, the computational speed suffers from intrinsic limitation and the usage of energy significantly increases [304].

Thus, in order to process and handle the datasets on a large scale proficiently, a novel computing architecture better than the traditional Von Neumann structure is required. Such a novel paradigm must be a simulated version of the human brain and must have the capability of performing complicated tasks even more efficiently than supercomputers. Therefore, it is quite evident that the neuromorphic computing field has gained the immense attention of researchers and is causing an overwhelming effect in the area of next-generation computing. The human nervous system comprises the activities performed together by the various intricate systems of networks available in the biological systems [305]. So, to emulate or surpass the human brain, an analogous multidisciplinary approach is needed in neuromorphic computing with the strenuous efforts from computing structure engineers, material scientists, circuit designers, device engineers, etc. To do this magnificent task, the synapse employed in the neural network is considered to be a stimulating factor. These neural network synapses can store information along with carrying out complicated tasks at the same place. This helps in the reduction of energy cost per operation by making networks capable enough of performing complex operations in a vastly parallel framework. Low-power neurons and spike-based computation are the reasons behind the ultra-high-energy competence of the human brain. Several neurons ($\sim$10$^{12}$) and synapses ($\sim$10$^{15}$) are available in the human brain and help in executing memory and learning functions [306]. Alteration in the synaptic strength, which is actually the synaptic weight of connections between different neurons, is responsible for the memorizing and learning functions, and this whole activity is known as synaptic plasticity [307–309]. Hence, the imitation of a bio-synapse is actually the process of implementing a human brain-motivated synaptic mechanism that has the capability of playing the role of an actual biological synapse [310, 311]. Artificial neural networks (ANNs), when compared with the classical computer system built using Von Neumann structure, possess advanced operational efficiency and less power consumption. Though, the conventional synaptic devices are not able to fulfil the requirements of ANNs due to some technological restrictions such as slower speed of response, higher consumption of power and large device area. In this context, RRAMs have become immensely promising candidates for designing artificial synaptic structures due to their capability of well imitating sundry biological synaptic activities and because of their modest metal/insulator/metal (MIM) assembly, low power consumption, large-density storage of data and rapid switching speed [312–315]. By applying an appropriate bias voltage to the RRAM device between its top and bottom electrode, it is possible to modulate its conductance. This can happen because of its flexible resistance switching characteristics between HRS and LRS. RRAM has a functional resemblance with biological synapse and thus acts as an artificial synapse in neuromorphic computing, and this incredible computational paradigm may fundamentally revolutionize the conventional Von Neumann computer device and outperform the modern computer architecture in data-intense applications [316–319].

### Basics of neural networks

A typical neural network consists of neurons and synapses which process the inputs from the previous layer and propagate it to successive layers after computing the weighted sum of inputs. Neural networks are employed to solve complex problems in the domain of deep learning, machine learning applications such as pattern and speech recognition. The hardware implementation of neural networks demands large storage memory for computing the vector matrix product, thus making computation more energy intense. In neural networks, the input and weights are processed and stored in the form of vectors; hence, the computation is also referred to as vector matrix multiplication [303]. Figure 26a depicts the biological neural network in which the biological neuron processes input information using dendrites and then transmits it to other neurons using synapses [320]. The artificial neural network equivalent of biological neural network depicted in Fig. 26b consists of several neuron layers interconnected via synapses. Figure 26c shows the crossbar array architecture implemented in hardware to obtain the vector matrix multiplication. The crossbar array stores conductance values of matrix in the memory cell, and these memory units mimic the functionality of the biological synapse. The application of suitable voltage pulse to the crossbar (rows and columns) enables selection of a particular voltage cell thus paving pathway for in-memory computation that is efficient in terms of area, power and latency [321].

RRAM devices can be utilized as synapses in providing the connection function between the information storage cells and neurons in the analog circuit of ANNs [309, 322–325]. The pivotal role of a synapse in a human brain is to transmit the impulses from one neuron to another during the process of information delivery in order to establish dynamic interconnections between two bonding neurons. The main function of an axon along with its terminals is to transmit the information out to other neurons (i.e. outputs), whereas dendrites are accountable for receiving the information (i.e. inputs). Triggering of a neuron releases a signal (pulse) that travels down the axon and into the dendrites of adjacent neurons via the synapses. The connection strength among the neurons or the synaptic weight decides the amount of signal that can reach the adjacent neuron. The small gaps (20–40 nm) between the axon terminal of a neuron and the dendrite of the other next neuron are referred to as synapses. As the brain starts adapting novel information, the synaptic weight becomes either stronger (potentiation) or weaker (depression) over the time through a process known as “synaptic plasticity”. The synaptic plasticity measured by the synaptic weight change is mainly distributed into long-term plasticity (LTP) and short-term plasticity (STP). The retention time of STP is from tens of milliseconds to few minutes, while LTP timescale can sustain over a few hours, even several days. In a biological neural system, STP lays the foundation for critical computation and LTP is considered to be responsible for the abilities in terms of memorizing and machine learning [326]. In addition to STP and LTP, the plasticity also varies with spike time. Spike-time-dependent plasticity (STDP) is the ongoing research area and is one among the innovative learning attributes in the human brain neuron system [327, 328]. STPD can be defined as a function relationship between change of synaptic weight and time interval resulting from activity variation of the pre- and post-neurons. The principle of synaptic plasticity is best described by Donald Olding Hebb, who proposed Hebbian theory in 1949, which specifies that synaptic transmission efficiency increases due to the continuous stimulation of presynaptic neurons to postsynaptic neurons. Hebb stressed that neuron “X” needs to make some contribution towards excitation of neuron “Y”, i.e. the excitation of the neuron “X” must precede the neuron “Y” and not simultaneously. This section of evaluation in Hebbian theory, later referred to as STDP (indicator of nervous system activity development), suggests that synaptic plasticity requires a certain time delay [329]. In the case of RRAM devices which are a type of memristor, the resistance states can be controlled by applied voltages because of the existence of RS characteristics and the microscopic conductive paths known as filament can also be acquired across the RS medium. Moreover, synaptic plasticity can be reproduced by running RRAM memory using unique coding strategies. As it is believed that the memorizing and learning tasks in the human brain are extensively dictated by the synaptic plasticity; thus, imitating the weight update process occurring during learning epochs is the crucial step in neuromorphic computing.

Synapses can be categorized as excitatory and inhibitory depending upon the type of neurotransmitter receptor on the synaptic membrane. Excitatory synapses correspond to excitatory postsynaptic potentials (EPSP), and postsynaptic neurons produce an action potential. On the other hand, inhibitory synapses produce an inhibitory postsynaptic potential (IPSP), and postsynaptic neurons do not reach the threshold for the formation of action potentials. The principles of postsynaptic inhibition and excitation are almost identical, as neurotransmitters binding to receptors open or close ion channels in the postsynaptic cell. The nature of postsynaptic response (EPSP or IPSP) is usually determined by channel type coupled to the receptor and the ion concentration of the cell.

### Pattern recognition using neural networks

**Fig. 27:**
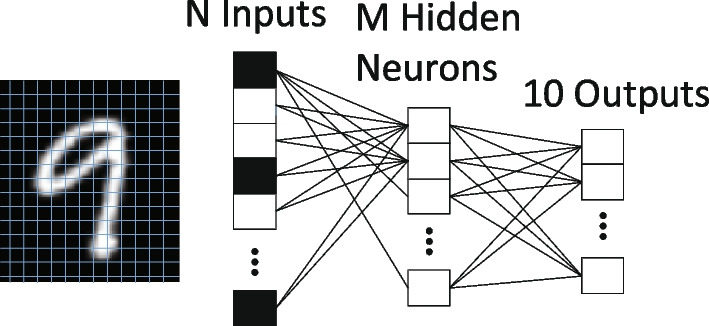
An illustration of pattern recognition for the case of handwritten digits recognition. The image is divided into N small clusters (i.e. N input elements) with all elements connected into a hidden neuron layer of size M, and those hidden neurons are then connected to the 10 output neurons, representing the digits 0–9. Reproduced with permission [297]. Copyright 2017, IEEE

For pattern recognition using neural networks, let us consider an example as demonstrated in Fig.  27 which highlights the process of computations performed by ANNs by a simple illustration of handwritten digits recognition using a fundamental feed-forward network. The primary image is divided into N black or white binary inputs, and each of these binary inputs corresponds to a particular image area. These input elements then implement a hidden layer where all the computations are carried out. Here, every binary input is linked to every M hidden neuron, in a similar way as every single hidden neuron is linked to all the 10 output neurons. All the inputs that are applied to a neuron are summed up and only the signal from that neuron propagates down to the next layer whose amount of input signal input has passed a SET threshold level. This process will carry out in every network layer until the signal reaches the output layer, thus 10 output neurons eventually correspond to the digits from zero to nine and the system can detect the corresponding digit in the primary image based on which neuron finally hits. The critical aspect in this overall process is regarding the decision that what should be the threshold signal level that an input neuron is required to hit and also about the measurement of synaptic weight (i.e. the strength of connections between the neurons). The connection strength or synaptic weight defines the amount of input signal that can be allocated to each neuron in the next layer. The learning procedure in the case of humans is somewhat complex, is dependent upon various diversified activities and thus is a separate subject under rigorous research analyses [297]. However, when artificial systems are under consideration, the training of these synaptic weights can be done via several algorithms of different complexity. One such conventional algorithm called offline learning is based on the introduction of a huge known data series into the system and then pertinent synaptic weights adjustment until the attainment of known correct output. Though this algorithm is effective, it suffers from some limitations such as the requirement of an already existing broad dataset that needs to be fed into the networks and a lot of time consumption during the process of training. Another technique known as online training involves the process of training the network with the introduction of data. This strategy is useful in the case of dynamic datasets but requires massive peripheral circuitry for performing huge weight update computations in real time and also for storing the new values of weights, large on-chip memory is needed [330].

Synaptic tools utilized in online training strategy require better endurance because of the on-the-fly updation of synapses. Moreover, such tools must have numerous conductance states having linear characteristics of weight change so that the network can easily meet the minimum error condition. Upon the completion of training, the network is capable of operating on its own to several units of success and this depends on the training schemes efficiency. Various online and offline training strategies are available to train the network, and they can be selected according to the type of network and what task is intended [331]. The networks are usually categorized into two major types: deep neural networks (DNNs) and spiking neural networks (SNNs). DNNs have proven their potential in several applications, for instance, in speech recognition and image classifications, whereas the field of SNNs is expanding and is a promising scheme in which biological neural systems are resembled scrupulously leading to maximum capabilities of ANNs.

### Current approaches and requirements for synaptic device

For the hardware execution of ANNs, numerous available neuromorphic mechanisms have been implemented. One of the DNN systems known as TrueNorth employs neurosynaptic cores based on 4096 CMOS. An overall 1 million neurons and around 256 million synapses are being formed by these cores which can perform 58 G-synaptic operations per second (GSOPS). Although its power efficiency is reasonable at $\approx$ 2.5 pJ op$^{-1}$, an improvement scope is there if compared with the efficiency of the human brain, which is around $\approx$ 2 fJ op$^{-1}$ [332]. On the contrary, SpiNNaker is among the SNN-based systems and employs 18 general-purpose CPUs and each CPU has the capability of modelling few hundred neurons [333]. Every neuron has synapses numbering in the range of 1000, though computing resources are significantly restricted by the software considerations. Further, all these systems are dependent on CMOS technology and thus suffer from power and area constraints leading to the limitations in the scaling up of sizes. To overcome all these shortcomings, researchers are vigorously putting their efforts into this area so as to come up with such designs of synaptic devices that can be efficiently employed for the hardware acceleration and implementation of ANNs.

Linear characteristics of conductance response, various operative states of conductance and extremely stable conductance for every state are the major requirements for synaptic devices [334–336]. Presently, the existing CMOS (complementary metal oxide semiconductors)-based synaptic devices available for the applications of the artificial intelligence consist of several capacitors and transistors for a single synapse, thus making the structure quite complex in case of mimicking plentiful synapses (10$^{15}$) present in the human brain [337, 338]. On the contrary, the demand for innovative synaptic and electronic devices has been augmented so as to surmount the cumulative transistor scaling cost and to overcome the inherent inefficiency that occurs because of the use of transistors in large quantities. In recent years, several synaptic paradigms with variable properties have been implemented. In the first glimpse, one can say that evolving nonvolatile memories (NVMs) share a lot more common properties with the synaptic devices; for instance, facilitation of programming is needed in both the mechanisms along with reading and information retention. Among various budding memory technologies useful in the nonvolatile synaptic applications, RRAM possessing the features of scalability, higher density and better chip design has proven to be an exceptional candidate. In comparison with the several types of synaptic methods, RRAM has been blessed with better endurance that lacks in FLASH memories, minimal size of the cell and less consumption of programming energy than MRAM (magneto resistive random access memory). Furthermore, the nonvolatile feature of RRAM overshadows the conventional DRAM which demands frequent recharging [339, 340].

**Fig. 28:**
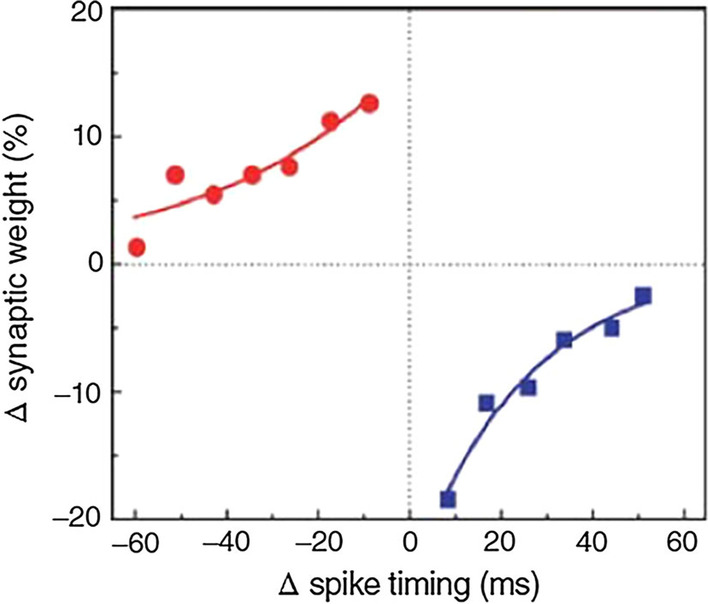
STDP curve of RRAM device based on experimental data. Reproduced with permission [347]

In the Synapse project headed by IBM researchers (TrueNorth), phase change memory (PCM) has revealed its potential to be used in the applications of neuromorphic computing [341]. But this PCM technology suffers from the limitation of higher consumption of power. Thus, one much touted and viable way out is the use of RRAM for such applications which has been analysed by Nanoelectronics and Nanotechnology Research Group from Stanford University and by NanoST laboratory from National Chiao Tung University [342, 343].The study by these research groups has revealed that RRAM has the capability of consuming much less power when executed as an artificial synapse and performs in a similar manner like that of PCRAM [344, 345]. Since an effective analysis by these groups, RRAM is in the limelight and has gained the immense interest of researchers belonging to various academic institutions worldwide. Particularly, oxide materials are involved in most of the RRAM studies. Oxide-dependent RRAMs are popular because of the CMOS-compatible, compact and simple structure of inherent metal insulator. Moreover, an important characteristic required to mimic adaptive synaptic variations is the multilevel behaviour that various oxide-based RRAM paradigms are capable of. Further, it is possible to lower the energy consumption per synaptic operation in the range of sub-pJ and the programming current can go further down to 1 $\mu$A [346]. Jo et al. in 2010 first demonstrated the RRAM device potential in synaptic applications [347]. An experimental RRAM STDP curve is illustrated in Fig. 28, and according to this curve, the increase in the conductance takes place with the time delay, and this occurs when the postsynaptic spikes are preceded by the presynaptic spikes (t<0). On the other hand, the conductance decreases when the presynaptic spikes are preceded by the presynaptic spikes (t>0). Along with the rise and fall in the synaptic weight, the synaptic weight variations could also be either short term or long term. Ohno et al. have shown both the short-term and long-term potentiation in $Ag_{2}$S-based RRAM device by causing the spike rate variations [348]. The results from this study have demonstrated that short-term potentiation behaviour has been exhibited by the RRAM device when pulse width at 0.5 s and inter-pulse delay of the 20 s is applied. This synaptic plasticity feature portrays a vital responsibility in the same way the brain realizes learning and memory. Despite the fact that the human brain performance simulation is a critical target to achieve, the literature about the present CMOS technologies suggested that the devices can surpass restrictions and can perform better and thus it becomes worth to mention that advancement and precision should be the ultimate goal. In such a scenario, strive for a technology that can outperform its biological counterpart should be there in which maximum reliability on device, maximum energy efficiency and speed of operation and linear and symmetric state switching can take place.

### RRAM-based prototype for neuromorphic computing

**Fig. 29:**
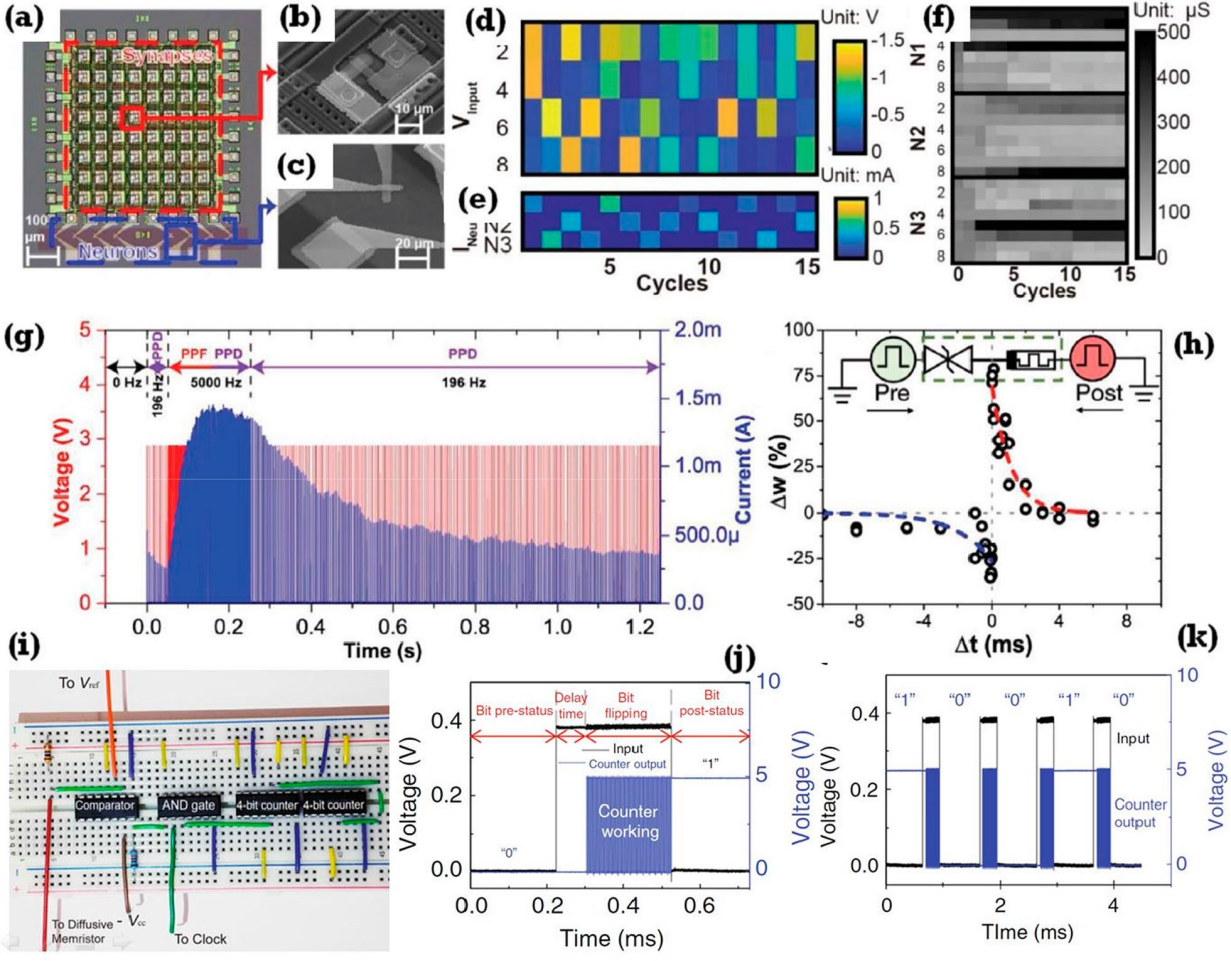
**a** Optical image of 8 × 8 RRAM-based neural network in 1T1R configuration. Scanning electron microscope image of **b** 1T1R cell and **c** a single 1R cell. **d** The input pattern, **e** peak neural current and **f** synaptic weight at each training cycle. **g** Experimental observation of short-term synaptic plasticity. **h** Conductance (weight) change of the drift memristor synapse in series with a diffusive memristor, as a function of the interval between pre and postsynaptic spikes, showing biorealistic STDP. Reproduced with permission from [352]. Copyright 2019 John Wiley and Sons Publishing. **i** Circuit arrangement of a RRAM device for TRNG application on bread board. **j** One counter output in response to 1 kHz input voltage pulse. **k** Random binary out flipping states over continuous switching cycles in the TRNG devices. Reproduced with permission from [353]. Copyright 2017 Nature Publishing Group

Traditionally RRAMs can fulfil the objectives of storage and memory devices. Analog or abrupt kind of switching occurs in RRAM. Such type of switching is quite significant in neuromorphic applications which require an accurate conductance change. To solve the problems related to artificial intelligence, integration of RRAM with CMOS technology can be proven to be very effective [349–351]. Neuromorphic computing architectures require a low-power and high-density structure having at least 5 bits/cell storage. A prototype of such neural network based on RRAM with 8 × 8 1T1R array in the Ag-doped ${\text{SiO}}_{x}{\text{N}}_{y}$ structure is shown in Fig. 29a–c [352]. The modulation of synaptic weight is demonstrated in Fig. 29d–f, and this has been achieved using a specific learning protocol and a peripheral circuit design. Ag-doped RRAM structures built using ${\text {MgO}}_{x}$, $SiO_{x}N_{y}$, and $HfO_{x}$ can help in mimicking both the long-term and short-term synaptic plasticity [354]. Figure 29g displays the paired-pulse measurement of such devices. Additionally, spike-timing-dependent plasticity (STDP) is demonstrated in Fig. 29h. Previously, many research works have identified the application of such RRAM devices for neuromorphic computing.

Since the times IOT-based devices have gained importance, the significant areas of applications of eNVM devices are in the domain of hardware security. For memory applications, it must be pointed out that stochasticity of eNVMs is not desired; rather, random state variations are preferable as entropy sources for security applications. In security applications, where STTRAM and RRAM are the key contenders, variability of eNVM devices in terms of random telegraph noise, resistance, switching voltage and switching yield controlled by operation conditions is important. For employing security systems based on RRAM devices, randomness is the key feature for various applications such as physical unclonable function (PUF) and true random number generator (TRNG). For these devices, the intrinsic stochastic nature is the significant source of entropy change (randomness), employed for generating random numbers and cryptographic keys. The cycle-to-cycle and device-to-device variations from LRS or HRS are used for realizing variations in a TRNG device. A volatile-type diffusive RRAM-based TRNG using the diffusion dynamics of metal atoms in Ag-doped $SiO_{2}$ structure is reported [353]. Figure 29i–k depicts the circuit arrangement consisting of an Ag-doped diffusive RRAM, a comparator, an AND-gate and a counter. The source of entropy for this case is considered to be the intrinsic stochasticity of the delay time. Figure 29i, j shows the experimental set-up using a simple circuit built on brad board and prototype working monitored using an oscilloscope, respectively. A train of pulses having constant amplitude ($V_{1}$ = 0.4 V) and pulse width of 300 and 700 $\mu$s spacing (i.e. 1 kHz frequency) was used. For the initial state, the bit was maintained at a low logic level (“0”), the counter started receiving clock signals (at 4 MHz) after some delay time and bit rapidly changed its state between low and high level (“0” and “1”). The counter stopped counting at the end of the input pulse ($V_{1}$), while maintaining its previous state until it will receive the next counting signal. The microcontroller reads this previous as the output bit. Due to the stochastic nature of the delay time for each cycle, the counter output after each pulse was totally unpredictable and flipped randomly between “0” and “1”. Figure 29k shows the monitored binary bits randomly flipped from “1”$\rightarrow$“0”$\rightarrow$“0”$\rightarrow$“1” $\rightarrow$“0” during four continuous ON-switching cycles.

### Neuromorphic computing utilizing 2D-materials-based RRAM

**Fig. 30:**
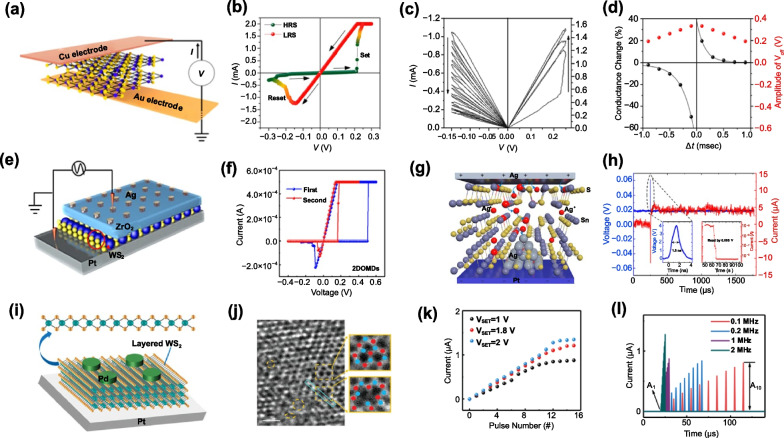
2D materials-based artificial synapse. **a** CF formation in Cu/$MoS_{2}$/Au-based RRAM. **b** I–V resistive switching characteristics and **c** switching characteristics of artificial synapse. **d** Depiction of STDP Behaviour. Reproduced with permission from [357]. Copyright 2019 Nano Letters. **e** Schematic of Ag/$ZrO_{2}$/$WS_{2}$/Pt-based RRAM, **f** I–V curve of Ag/$ZrO_{2}$/$WS_{2}$/Pt-based RRAM. Reproduced with permission from [358]. Copyright 2019, American Chemical Society. **g** Depiction of ion dynamics in Ag/SnS/Pt RRAM. **h** Demonstration of faster switching times with 4 V/1.5 ns voltage pulse. Reproduced with permission from [359]. Copyright 2021, Nano Letters. **i** Atomic vacancy formation and migration in Pd/$WS_{2}$/Pt RRAM device. **j** TEM image of the RRAM device in the LRS. **k** Pulse modulation of the device obtained by varying amplitude of the input voltage. **l** Current response for the train of pulses with varying frequencies. Reproduced with permission from [360]. Copyright 2019 Small

The use of 2D materials for realization of RRAM device enables device scaling to 10 nm thickness or less, which ensures switching at lower voltages which is very useful for low-power applications [355, 356]. Xu et al. demonstrated a bilayer RRAM structure based on $MoS_{2}$ switching layer with schematic depicted in Fig. 30a [357]. The bipolar switching behaviour of the device is observed with small set voltage of 0.2 V, which is mostly attributed to the electrochemical metallization of Cu ions as depicted in Fig. 30b. The device can be used for artificial synapse applications due to the analog resistance switching characteristics. Figure 30c illustrates the gradual conductance change observed in the device due to the variation of the CF width with varying DC bias voltages. The device also exhibited STPD characteristics with multi-step potentiation and depression of synaptic weights as depicted in Fig. 30d, making it suitable choice for artificial synaptic devices in neuromorphic systems.

In RRAM devices, random nature of the CF formation and rupture significantly affects the device performance primarily in terms of stability and uniformity. Yan et al. demonstrated RRAM device with heterojunction composed of $ZrO_{2}$/$WS_{2}$ layers for controlled CF formation and rupture with schematic shown in Fig. 30e [358]. The switching properties of the device outperform the single-layer devices due to the limited rupture and regeneration of CFs in the bilayer region as depicted in Fig. 30f. Lu et al. demonstrated a RRAM device with Ag/SnS/Pt structure shown in Fig. 30g that had robust CF formation primarily due to the Sn vacancies [359]. The presence of Sn vacancies is deemed to be more favourable in terms of energy efficiency for migration of metal ions compared to chalcogen vacancies as it allows for low energy and ultra-fast switching. The Ag/SnS/Pt RRAM device was reported to demonstrate great performance with various performance metrics such as faster switching times of 1.5ns or less, low switching voltage of 0.2 V and lower power consumption of less than 100 fJ as shown in Fig. 30h. Yan et al. reported Pd/$WS_{2}$/Pt RRAM device in which the resistive switching occurred without the filament formation due to the vacancy formation and electron hopping across the vacancies as depicted in Fig. 30i [360]. It was observed through TEM examination that a larger number of S and W vacancies in the LRS of the $WS_{2}$ nanosheet were present compared to the pristine one as shown in Fig. 30j. Due to the application of voltage pulse, the temperature increases internally due to the Joule heating and thermophoresis effect, which causes increased vacancies to be generated in S and W sites. This results in the rise of the overall conductance, as defects are increased at LRS and electron hopping also increases due to the shorter distances between them. Figure 30k, l shows numerous synaptic plasticities (spiking voltage, spiking frequency and spiking width dependent plasticity) emulated utilizing various pulse programming methods. The gradual increase in the device current is observed on the application of pulse sequence as input; eventually, a point is observed where the current reaches the upper limit. A larger saturation current (with 2 V pulse) demonstrates better spiking voltage-dependent plasticity characteristics as compared to the 1 V pulse. Also low device current is observed in these modulations, resulting in low power consumption which is very desirable for neuromorphic applications.

## Summary and outlook

During last couple of decades, significant efforts have been made in the domain of RRAM technology primarily with focus on research and development sector, with aims to commercialize it and understand it. As of today, its adoption is still limited, and its understanding is still incomplete. RRAM technology offers many unique properties worth the research and development efforts, as well as helps overcome difficult scaling barriers. In this work, we have provided an overview of RRAM devices with advances in various fields including thin-film materials applied in RS layer and electrode, classification of RS mechanisms and investigation on artificial synapse. The fabrication of devices based on RRAM has been reported in various research works using inorganic materials, such as oxides, solid electrolyte and 2D materials, with relatively mature performance demonstrated by the devices. Thus, the RRAM devices possess a great scope for the application of organic materials. The performance of the devices depends largely on the RS mechanisms, which also has a strong connection with choice and processing techniques of the thin-film materials. RRAM technology based on different materials has shown a great potential in terms of large-scale commercialization which is quite promising. In addition to the traditional large-scale commercialization process, the larger objective of analysing different RRAM device performances is to provide potential assistance to artificial intelligence and neuromorphic computing systems. RRAM devices can mimic functions of biological synapse with electrical performance, which has a positive influence in hardware application of the artificial intelligence field. In addition, its human-brain-like behaviours such as STM and LTM make the development of neuromorphic computing system possible in the coming future. Although there is still a long way until mass application and industrialization of RS devices, as research goes on, novel low-dimensional nanomaterials have been proven to be a promising candidate with fewer side effects on performance improvement, and will also guarantee the fabrication of small dimensional device. With the coming of big data era, there are huge demands on 3D integration of memory array, neuromorphic computing and transparent flexible device. Low-dimensional nanomaterials will undoubtedly play an irreplaceable part in such fields in the future.

## Data Availability

Not Applicable.
